# Associations Between Patient Health Outcomes and Secure Message Content Exchanged Between Patients and Clinicians: Retrospective Cohort Study

**DOI:** 10.2196/19477

**Published:** 2020-10-29

**Authors:** Dawn M Heisey-Grove, Laura E McClelland, Cheryl Rathert, Alexander Tartaglia, Kevin Jackson, Jonathan P DeShazo

**Affiliations:** 1 MITRE Corporation McLean, VA United States; 2 College of Health Professions Virginia Commonwealth University Richmond, VA United States; 3 Department of Health Management and Policy Saint Louis University St Louis, MO United States; 4 Department of Nursing and Allied Health College of Science, Engineering, and Technology Norfolk State University Norfolk, VA United States

**Keywords:** health information technology, electronic messaging, patient physician communication, diabetes, hypertension

## Abstract

**Background:**

The number of electronic messages securely exchanged between clinic staff and patients has risen dramatically over the last decade. A variety of studies explored whether the volume of messages sent by patients was associated with outcomes. None of these studies, however, examined whether message content itself was associated with outcomes. Because secure messaging is a significant form of communication between patients and clinic staff, it is critical to evaluate the context of the communication to best understand its impact on patient health outcomes.

**Objective:**

To examine associations between patients’ and clinicians’ message content and changes in patients’ health outcomes.

**Methods:**

We applied a taxonomy developed specifically for secure messages to 14,394 patient- and clinic staff–generated messages derived from patient-initiated message threads. Our study population included 1602 patients, 50.94% (n=816) of whom initiated message threads. We conducted linear regression analyses to determine whether message codes were associated with changes in glycemic (A1C) levels in patients with diabetes and changes in systolic (SBP) and diastolic (DBP) blood pressure in patients with hypertension.

**Results:**

Patients who initiated threads had larger declines in A1Cs (*P*=.01) compared to patients who did not initiate threads. Clinic nonresponse was associated with decreased SBP (β=–.30; 95% CI –0.56 to –0.04), as were staffs’ action responses (β=–30; 95% CI –0.58 to –0.02). Increased DBP, SBP, and A1C levels were associated with patient-generated appreciation and praise messages and staff encouragement with effect sizes ranging from 0.51 (A1C) to 5.80 (SBP). We found improvements in SBP associated with patients’ complaints (β=–4.03; 95% CI –7.94 to –0.12). Deferred information sharing by clinic staff was associated with increased SBP (β=1.29; 95% CI 0.4 to 2.19).

**Conclusions:**

This is the first research to find associations between message content and patients’ health outcomes. Our findings indicate mixed associations between patient message content and patient outcomes. Further research is needed to understand the implications of this work; in the meantime, health care providers should be aware that their message content may influence patient health outcomes.

## Introduction

### Background and Significance

The use of secure messaging—email messages exchanged between patients and clinical staff through a secure platform—has increased significantly over the last 2 decades as patients’ access to the functionality increased [1-5]. Patients reported that secure messaging offered convenience, with the added benefit of documenting the conversation so that it could be referenced later [6-9]. Although clinicians cited challenging workflows as the biggest barrier to use [10], they noted that secure messaging improved communication between visits and boosted patient engagement, satisfaction, and trust [7,9].

Communication between patients and clinicians should include information exchange, uncertainty management, relationship development and fostering, and activities that enable decision making and health self-management [11]. According to Street et al [11], these communication functions lead to proximal and intermediate outcomes that eventually result in improved patient health outcomes. Prior research provides evidence that secure messaging is a significant modality for patient–clinician communication [4,12,13]. As such, message threads that include communication functions identified by Street et al [11] should be associated with better health outcomes.

To the authors’ knowledge, there is no published research to date that linked secure message content and patients’ health outcomes. Rather, researchers have explored whether the number of secure messages, or secure message use more generally, was associated with outcomes for a variety of conditions. An equal number of studies found that secure message use was associated with improvements in blood pressure control [14-16], or no association between them [12,16,17]. While a number of studies identified positive associations between secure message use and controlled glycemic levels [12,14,17-19], 2 studies identified no association [18,20], and 1 found inconsistent associations [16]. Even within studies, findings varied across conditions. For example, Price-Haywood et al [16] assessed performance on population-level measures for glycemic and blood pressure control and found that both improved among some patients with diabetes with evidence of a dose response. This did not hold, however, among patients with diabetes who had glycemic levels over 8%, nor for blood pressure changes among patients with hypertension. Similarly, Harris et al [18] found that the highest users of secure messaging had better glycemic control but did not identify similar patterns with blood pressure control. An analysis by Shimada et al [17] separated prescription refill requests and other types of secure message use and found that improvements in glycemic control after 2 years were associated with secure message use but not with prescription refills. Interestingly, they found the reverse was true for blood pressure control. These mixed findings, particularly the findings from Shimada et al [17], suggest that we must move beyond counting messages and begin classifying and quantifying message content types in order to better understand how secure messages, and more specifically, secure messaging content, impact important patient health outcomes. This research study directly attempts to address this need.

### Objective

We created and applied a theory-based taxonomy developed specifically for secure messaging to a large sample of patient- and clinician-generated messages and explored whether certain types of message content were associated with changes in glycemic levels among patients with diabetes and changes in blood pressure among patients with hypertension [21,22]. Our taxonomy provides taxa (ie, codes) for patient- and clinic staff–generated content. Multimedia Appendix 1 includes a complete list of taxa (ie, codes), their definitions, and examples of each taxon. We included taxa (italicized throughout this paper) to classify patient-generated *Information seeking* and *Information sharing* (eg, *Self-reporting* and *Clinical updates*), and *Task-oriented requests* such as *Appointment scheduling*, *Prescription refill requests*, and *Other administrative requests*. We used different taxa to classify clinic staff–generated *Information sharing* and *Action responses* that indicate the degree of request fulfillment, and *Information seeking*. Finally, we classified *Social communication* that may be related to fostering relationships and trust among messages generated by either party.

Based on Mishel’s Uncertainty in Illness Theory [23] and Street et al’s [11] framework, we anticipated that actions indicative of self-care, such as *Task-oriented requests* not associated with uncertainty (eg, routine appointment requests, prescription refills) and *Self-reporting*, would be associated with improved health outcomes. Similarly, actions from the patients that might be indicative of trust between patient and clinician, such as *Information sharing* and positive *Social communication*, would be associated with improved health outcomes. Clinicians’ *Information sharing* and *Encouragement* could be a mechanism to mitigate patients’ uncertainty and improve trust, so we hypothesized that receipt of this message content would also result in improved health outcomes. Conversely, we anticipated that clinician responses that did not mitigate patients’ uncertainty would result in poorer outcomes. We included in this category lack of a response from clinic staff to patient-initiated threads and clinic staffs’ *Information sharing/Deferrals*. Finally, we expected that patients who expressed negative *Social communication* (eg, *Complaints*) would also experience poorer health outcomes.

We present this nascent research to provide early evidence that the content of some secure messages between patients and clinic staff may be predictive of outcomes for patients with chronic illness.

## Methods

### Study Population

Our study population included adult patients who registered with the patient portal of a large urban academic medical center. The portal allowed registered patients to send and receive secure messages with clinic staff, request appointments and prescription refills and renewals, view upcoming appointments and notes from prior visits, and find links for health-related educational materials and bill pay. Patients could access the portal through any device with a web browser.

Patients had to have at least one inpatient or two outpatient visits in 2016 with ICD-10-DM diagnosis codes for either diabetes (E11) or hypertension (I10), and one visit in 2018. We stratified the sample based on health condition (diabetes only, hypertension only, or both conditions) and whether patients initiated a message thread between January 1 and December 31, 2017, then randomly selected samples from each stratum. Patients who lacked baseline or endpoint values for the outcomes of interest were excluded from this study.

We included 2 different conditions in our research to control for disease condition, not to provide specific recommendations relative to condition. Our message sample included all patient-initiated threads generated by those sampled patients and saved to patients’ charts between January 1 and December 31, 2017. We included only patient-initiated threads because we felt these were the best markers of patient uncertainty and self-management. This research received approval from the VCUHS Institutional Review Board under expedited review. We manually extracted messages from patients’ electronic health records and redacted all identifiable information during the extraction process. We coded those deidentified messages, which were linked with a unique identifier not linked to the patients’ medical record.

Our study sample consisted of 1602 patients (full population), of whom 50.94% (n=816) initiated at least one message thread (secure message-only population). We included patients with diabetes only (n=347), hypertension only (n=751), and both conditions (n=504). We coded 5844 message threads initiated by these patients, which included 8008 patient-generated messages and 6386 messages generated by 496 unique clinic staff. Our sampled population generated an average of 9.81 messages (median 5; max 117). Message responses to patient-initiated messages came from physicians, nursing staff, administrative staff, pharmacists, physician assistants, medical assistants, podiatrists, social workers, and medical technicians from departments and clinics across the medical center. Clinics employed a triaging system whereby administrative or nursing staff review incoming patient-generated messages and determine the best response approach. Decisions about which staff would serve as the triage point, and the triaging process itself, varied across clinics.

### Independent Variables

Table 1 lists the taxa, or codes, used in these analyses. Multimedia Appendix 1 provides the taxa definitions and examples for each. We created the taxonomy by leveraging common taxa reported in other published literature. Our 2 patient-generated *Information seeking* taxa were selected based on Mishel’s Uncertainty in Illness Theory [23], which identifies the reasons patients might outreach to clinicians to manage their uncertainty around their illness. We included *Information seeking* and *Information sharing* taxa for both patients and clinicians in recognition that information exchange is a communication function on the pathway to improved patient outcomes [11]. In addition, we included other constructs from the Street et al [11] pathway, including *Task-oriented* patient-generated requests that might be markers of patient self-management, and *Social communication*. We leveraged the Taxonomy of Requests by Patients [24] for clinic staff-generated *Action responses* to those patient requests. We piloted our taxonomy with a small sample to ensure that no constructs were missing and that the appropriate level of granularity was present in the taxa [22].

We used the taxa to distinguish between different types of patient-generated and clinic staff–generated message content. For these analyses, we report findings for the individual taxa as well as the level 1 groupings of taxa for patient *Information seeking*, patient *Information sharing*, patient *Social communication*; patient *Task-oriented requests* reflective of self-management; other patient *Task-oriented requests*; staff *Information sharing*; and staff *Action responses*. We based our independent variables on counts of taxa either sent or received by patients between January 1 and December 31, 2017. Because we found a strong correlation between the likelihood of sending and receiving a taxon based on patients’ thread volume, our independent variables measure taxa as a function of volume. Each taxon is represented in the linear regression models as a proportion of the total patient-generated or clinic staff–generated taxa they sent or received.

We also created an independent variable that measured clinic nonresponse, defined as a thread that included no messages sent from clinic staff. We measured clinic nonresponse as a proportion of the total threads initiated by the patient.

**Table 1:** Secure message taxonomy.

Patient or clinician generated taxon, Level 1, and Level 2 taxon			Level 3 taxon
**Patient generated**			
	**Information seeking**		
		Logistics	N/A^a^
		Medical guidance	N/A
	**Information sharing**		
		Clinical update	N/A
		Response to clinician’s message	N/A
		Self-reporting	N/A
	**Task oriented**		
		Prescription refills and requests	N/A
		New or change prescription request	N/A
		Other administrative	N/A
		Referral requests	N/A
		Scheduling request	Cancellation, Follow-up, Laboratory test or diagnostic procedure, New condition or symptom, Preventive care or physical examination, Reschedule
	**Social communication**		
		Appreciation or praise	N/A
		Complaints	N/A
		Life issues	N/A
**Clinic staff generated**			
	**Action responses**		
		Acknowledge	N/A
		Denies	N/A
		Fulfills request	N/A
		Partially fulfills request	N/A
	**Information seeking**		
		N/A	N/A
	**Information sharing**		
		Deferred	N/A
		Medical guidance	N/A
		Orientation to procedures, treatments, or preventive behaviors	N/A
	**Task oriented**		
		Recommendation to schedule appointment	N/A
	**Social communication**		
		Encouragement	N/A

^a^N/A: Not applicable.

### Dependent Variables

We created 1 dependent variable for patients with diabetes and 2 for patients with hypertension. For patients with diabetes, we measured the change between the endpoint and baseline measures of glycemic control (A1C). For patients with hypertension, we included dependent variables that measured changes between baseline and endpoint measures for systolic blood pressure (SBP) and diastolic blood pressure (DBP). We used the last recorded value in 2016 as the baseline measure. Our endpoint value was the first measured value obtained between January and June 2018. If multiple blood pressures were taken on the same day, we averaged available values.

### Covariates

Based on prior literature relating to patient–clinician communication and electronic communication practices [25-29], we expected differences in taxa use based on patient and clinician characteristics. We therefore controlled for patient age as of January 1, 2017; patient sex; race (Black, White, and other); payer type (public, private, uninsured, or other); rural home location as a bivariate derived from Rural–Urban Commuting Area codes [30]; health condition (diabetes only, hypertension only, or both conditions); the number of outpatient and inpatient visits during 2017; and the number of threads initiated during 2017. We also included baseline A1C and blood pressure values in models measuring change in glycemic control and blood pressure, respectively. For models that included only patients who initiated message threads (ie, secure message–only population), we included the average distance between zip code centroids of patients’ homes and the clinics to which they sent messages.

### Qualitative Analyses

We assigned taxa to all messages—those generated by patients and clinic staff—that were saved to the patient’s chart and part of patient-initiated threads created and completed between January 1 and December 31, 2017. Our context unit was the message thread. Coding units were no longer than a single message but could be shorter depending on the content in the message (eg, if multiple taxa are applied to the message). A given message was assigned as many taxa as there were concepts in the message; however, we limited the number of times a given taxon (ie, a single code) could be counted for each message to 1 per message.

A primary coder (DH-G) assigned taxa to all messages while a secondary coder (JDS) applied taxa to a random sample of messages (n=1908). The primary coder trained the secondary coder based on a set of definitions and sample coded text collected from a pilot study [22]; these samples and definitions were refined as the coding process continued. We conducted the coding in batches and discrepancies were discussed and reconciled following the completion of each batch. The primary coder recoded each batch as appropriate based on those discussions. Multimedia Appendix 2 lists the interrater and intrarater reliability coefficients for the last coded batch. Intrarater reliability ranged from fair to excellent. Three taxa received a poor kappa rating when comparing the results from the 2 coders (interrater reliability): clinician-generated *Action response/Denies*, *Recommendation to schedule*, and patient-generated *Information seeking/Logistics*.

### Quantitative Analyses

We estimated unadjusted differences by patient characteristics based on use of secure messaging by applying chi-square analyses for categorical variables and unpaired *t* test of means for continuous variables. We executed 2 linear regression analyses for each combination of taxon and dependent variable. The first model used the full population and the second used the secure message–only population. The comparison in the full population models included all patients who did not initiate a message thread and those patients who sent or received messages with the selected taxon. Models that included the secure message–only population compared patients who sent or received messages coded with the selected taxon to those who sent or received other types of messages. We report results as the unstandardized regression coefficient (β weight). We conducted all analyses using SAS version 9.4 (SAS Institute).

## Results

Table 2 presents the descriptive statistics for our study population, comparing the populations who sent messages in 2017 to those who did not. Among patients with diabetes, we observed differences in the use of secure messaging by age, condition, insurance type, and sex. Except for age, we observed similar differences in use of secure messaging among patients with hypertension.

**Table 2:** Comparison of study population’s characteristics by use of secure messaging in 2017.^a,b^

Characteristics		Patients with diabetes			Patients with hypertension		
		Sent messages (N=430)	Did not send messages (N=421)	*P* value	Sent messages (N=621)	Did not send messages (N=634)	*P* value
Age in years, mean		57.84	59.80	.02	59.97	58.65	.08
Distance between home and clinic in miles, mean		27.48	N/A^c^	N/A	33.47	N/A	N/A
**Conditions**							
	Both, n (%)	235 (54.7)	269 (63.9)	.006	235 (37.8)	269 (42.4)	.10
	Diabetes only, n (%)	195 (45.3)	152 (36.1)	.006	N/A	N/A	N/A
	Hypertension only, n (%)	N/A	N/A	N/A	386 (62.2)	365 (57.6)	.10
**Home location**							
	Rural, n (%)	9 (2.1)	17 (4.0)	.10	17 (2.7)	29 (4.6)	.08
	Urban, n (%)	421 (97.9)	404 (96.0)	.10	604 (97.3)	605 (95.4)	.08
**Insurance**							
	Other, n (%)	126 (29.3)	93 (22.1)	.02	160 (25.8)	160 (25.2)	.83
	Private, n (%)	138 (32.1)	80 (19.0)	<.001	155 (25.0)	110 (17.4)	<.001
	Public, n (%)	161 (37.4)	241 (57.2)	<.001	296 (47.7)	349 (55.0)	<.001
	Uninsured, n (%)	5 (1.2)	7 (1.7)	.54	10 (1.6)	15 (2.4)	.34
**Race**							
	Black, n (%)	193 (44.9)	214 (50.8)	.08	231 (37.2)	316 (49.8)	<.001
	Other, n (%)	24 (5.6)	27 (6.4)	.61	23 (3.7)	26 (4.1)	.72
	White, n (%)	213 (49.5)	180 (42.8)	.05	365 (58.8)	291 (45.9)	<.001
**Sex**							
	Male, n (%)	134 (31.2)	182 (43.2)	<.001	235 (37.8)	279 (44.0)	.03
	Female, n (%)	296 (68.8)	239 (56.8)	<.001	386 (62.2)	355 (56.0)	.03

^a^Percentages represent the proportion of the population with that characteristic.

^b^The *P* value is the unadjusted estimate of statistical difference between the populations who sent secure messages and those who did not.

^c^N/A: Not applicable.

### Change in A1C Among Patients With Diabetes

Among patients who initiated threads, we observed a mean A1C decrease of –0.56, with the 2018 value being statistically lower on average than the 2016 value (*P*<.001). The same was not true among patients who did not initiate threads (*P*=.20). We observed differential changes between 2016 and 2018 when comparing patients with a baseline A1C indicating controlled diabetes (<7.0) versus uncontrolled diabetes (>7.0). Among patients who sent secure messages, we observed a mean increase in A1C of .04 for patients with controlled diabetes (n=151), compared to a mean decrease of –1.22 among patients with uncontrolled diabetes (n=138).

**Figure 1:**
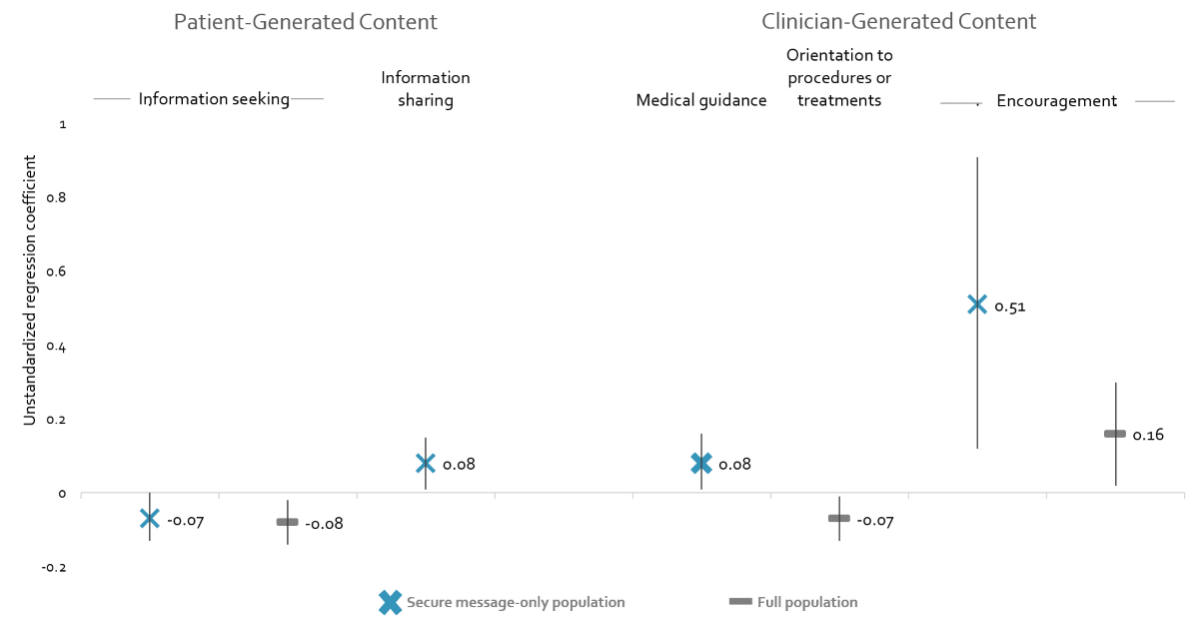
Associations between taxa and A1C changes.

Figure 1 displays statistically significant associations between taxa and A1C changes for the taxa. Taxa not represented in the table were not associated with A1C changes at *P*<.05 but are available in Multimedia Appendix 3, which also lists the *P-*value for all covariates. Baseline A1C was the only covariate with statistical significance (*P*<.001) across all analyses.

As their proportion of *Information seeking* increased, patients experienced greater declines in their A1C values. This was true when comparing patients within the full population and among the secure message–only population. Conversely, patients who shared information with their clinic staff experienced A1C increases, compared to those who sent other types of messages to clinic staff. Three clinician-generated subtaxa were associated with A1C changes. Patients who received *Orientation to procedures and treatments* had declines in A1C compared to patients who did not initiate threads (β=–.07; 95% CI –0.13 to –0.01), whereas patients who received *Medical guidance* had increased A1C compared to patients who received other content from clinic staff (β=.08; 95% CI 0.01-0.16). Similarly, patients who received *Encouragement* from clinic staff experienced increased A1C values (β=.16; 95% CI 0.02-0.03) between 2016 and 2018.

### Change in SBP Among Patients With Hypertension

Overall, we observed an unadjusted average increase in SBP between 2016 and 2018 among patients who initiated threads (3.41-point increase; *P*<.01) and patients who did not initiate threads (2.45-point increase; *P*=.02). Among patients who sent secure messages, those with controlled systolic blood pressure (<120 mmHg) experienced a mean increase in SBP of 16.41 (n=149), compared to a mean decrease of –0.71 among those with uncontrolled SBP (n=471).

**Figure 2:**
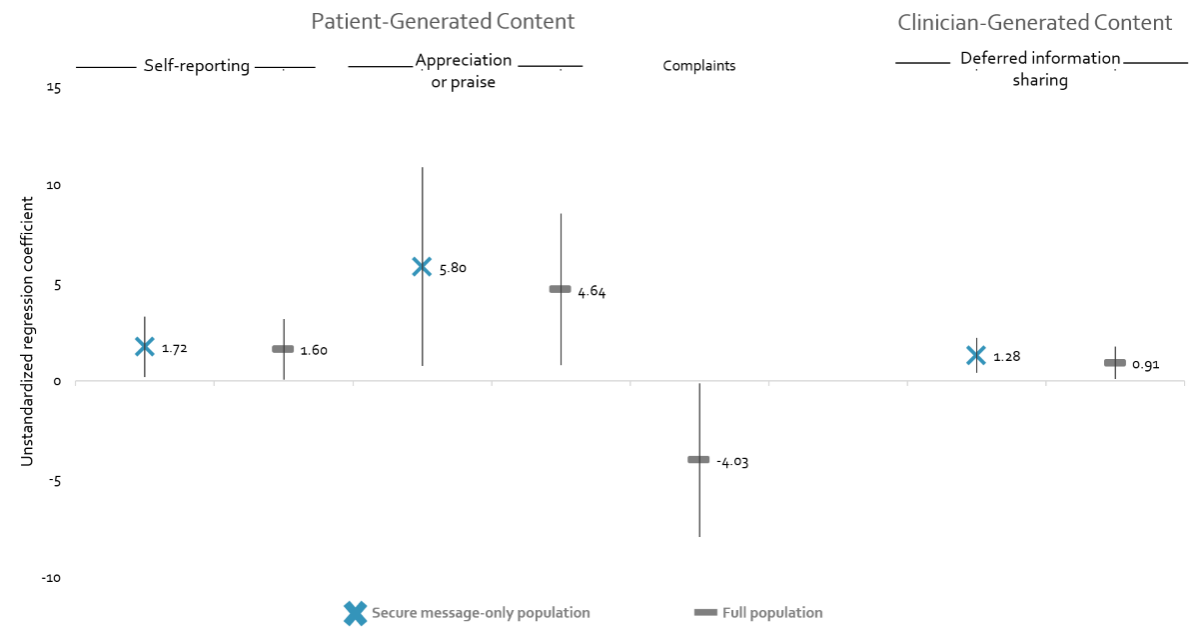
Associations between taxa and SBP changes. SBP: systolic blood pressure.

Figure 2 presents the taxa associated with SBP changes among these populations. Full analysis results are available in Multimedia Appendix 3. Two patient-generated taxa (*Self-reporting* and *Appreciation or praise*) and the clinician-generated taxon for *Information sharing/Deferral* were associated with increased SBP. This was true in both population comparisons. By contrast, we observed decreased SBP among patients who sent *Complaints* compared to patients who did not initiate threads. Covariates of statistical significance included baseline SBP (*P*<.001), race (Black vs White; *P*<.05), and, for some analyses, age (*P*<.05) and number of outpatient visits (*P*<.05).

### Change in DBP Among Patients With Hypertension

Among patients who initiated threads and those who did not, we found statistically significant unadjusted increases in DBP between 2016 and 2018 of 1.71 and 1.67, respectively (*P*=.007 and *P*=.003, respectively). Among patients who sent secure messages, patients with controlled DBP (n=351) at baseline (<80 mmHg) experienced a mean increase in DBP of 6.57, compared to a mean decrease of –4.62 among patients with uncontrolled DBP (n=269) at baseline (>80 mmHg).

**Figure 3:**
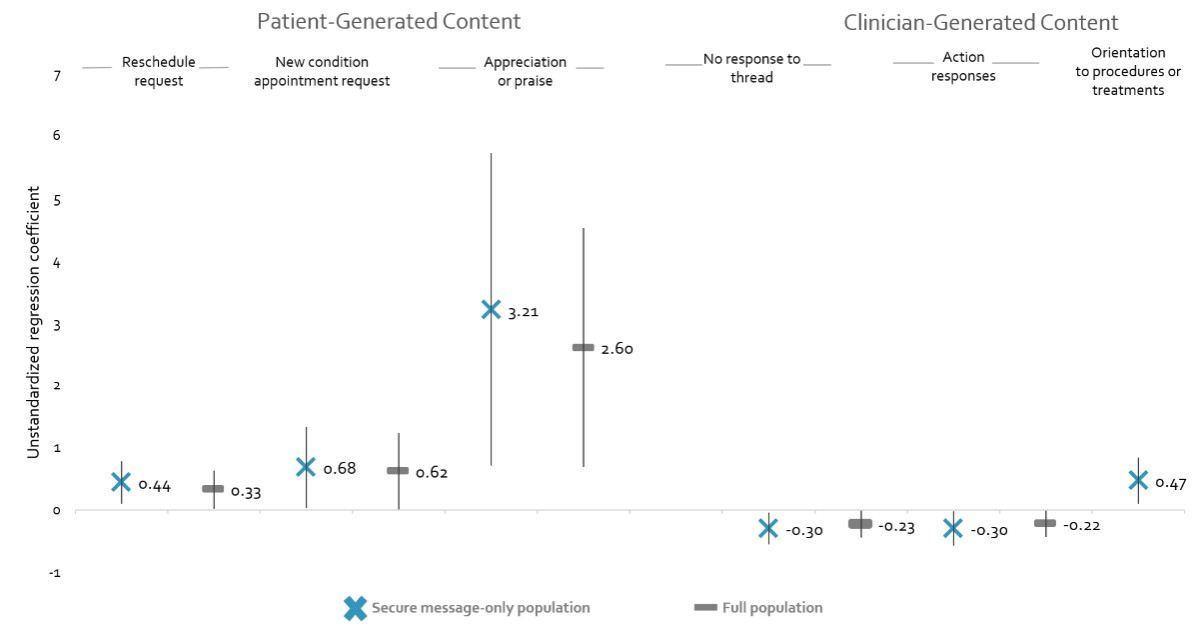
Associations between taxa and DBP changes. DBP: diastolic blood pressure.

Figure 3 presents the associations between taxa and changes in DBP that were statistically significant at *P*<.05. Full analysis results are available in Multimedia Appendix 3. Three covariates were statistically significant across all analyses (age [*P*<.01], baseline DBP [*P*<.001], and number of outpatient visits [*P*<.05]).

Three patient-generated taxa were associated with increased DBP (*Schedule request/Reschedule,* *Schedule request/New symptoms or conditions*, and *Appreciation or praise*). Similar effect sizes were observed across the 2 models for each taxon. We also observed that as the prevalence of clinic staffs’ nonresponse increased, patients experienced greater declines in DBP. Similarly, patients who received proportionally more *Action response*–related content (including *Acknowledgment*, *Fulfills request*, and *Partially fulfills request*) had greater DBP decreases. Patients who received *Orientation to procedures or treatments* had correspondingly increased DBP, compared to patients who did not receive those kinds of messages from clinic staff.

## Discussion

### Principal Findings

We present here the first findings exploring associations between secure message content and patient health outcomes. Our research found associations between selected message content and changes in patients’ glycemic levels and blood pressures. As we anticipated, patients who sent *Information seeking* messages, or who received *Orientation to procedures or treatments* messages from clinic staff, experienced greater decreases in A1C. Consistent with other research [12,14,16-19], we found an overall association between secure messaging use and improved A1C. Also as expected, we observed DBP decreases among patients who received confirmation of action, SBP increases in response to *Information sharing*/*Deferral*, and DBP increases associated with *Scheduling request/New condition*.

Counter to our hypotheses, however, we found that A1C increased among patients who shared information with clinic staff. We were only able to identify a statistically significant association in the level 1 *Information sharing* taxon (*P*=.02). Associations in the *Information sharing* subtaxa were not statistically significant (*P*>.05). Detecting an association at the grouped *Information sharing* level makes it challenging to interpret these results, as patients who provide *Clinical updates* may be receiving care from multiple providers and may have other health conditions that impact their glycemic levels. Patients who self-report to their clinicians, however, may be practicing self-management of their condition which, according to Street et al [11], should be associated with improved patient health outcomes. Similarly, information exchange, as represented in the *Information sharing/Response to clinician’s message*, should be associated with improved outcomes. Our inability to detect a statistically significant association at the subtaxa level for *Information sharing* makes it difficult to interpret these results further. Future studies should consider expanding the sample size to improve the ability to detect associations at the subtaxa level.

We also observed DBP increases associated with certain types of patient-generated *Scheduling requests* and *Information sharing* by clinic staff. It could be that these patients had new procedures or other treatments that contributed to their DBP increases which could be a confounder to any possible influence on outcomes. Although our analyses controlled for patients’ number of outpatient and inpatient visits, we did not control for severity of co-occurring conditions or types of procedures patients may have undergone during the study period. We did find that baseline DBP was a significant (*P*<.001) predictor of the patients’ change in DBP between baseline and endpoint.

We also observed an inverse association between DBP and clinic nonresponse: patients’ DBP decreased as nonresponse prevalence increased. Our findings should not be taken as advocating for nonresponse to improve patients’ health, because there are many unanswered questions about what is driving these findings. It will be important to assess when nonresponse may be appropriate and when it might have deleterious effects. For example, it is possible that certain types of patient-initiated threads do not require a clinic response, such as when a prescription refill request is completed, and patients are notified by their pharmacy that the prescription is ready rather than the clinic. Future research should explore the best communication modality for responses.

In the analyses that used only patients who initiated message threads, we found that as the prevalence of *Schedule request/Reschedule* increased, so did patients’ DBP. We interpreted a reschedule request as a manifestation of self-care, following Mishel’s Uncertainty in Illness Theory [23], because patients are taking charge of their health care visits by rescheduling to a time more convenient to them, thereby leading to less stress. Our findings indicate this is not the case. Instead, the poorer outcomes associated with *Schedule request/Reschedule* may be a manifestation of stress in patients’ lives that required rescheduling medical appointments, a lack of or decline in activation, or a deficit in access to care (eg, transport issues). If these patients were not managing their stress and not maintaining their levels of self-care, their health outcomes might suffer as their patient activation threshold declined [31-33]. Consistent with this, we observed similar poor outcomes—with slightly larger effect sizes—associated with appointment requests for new conditions or symptoms.

Our research identified different effects by health condition on outcomes associated with staff sharing *Orientation to processes and treatments*. Patients with diabetes who received these messages had lower A1C levels in 2018 but patients with hypertension experienced increased DBP. Clinician-generated *Information sharing/Deferral* was associated with increased SBP among patients with hypertension, but we did not observe similar associations for A1C changes. It will be important to apply this taxonomy to other conditions to determine if other differences between outcomes and communication content exist by condition, to better improve communication between patients and clinic staff in ways that advance patients’ health.

We included a number of covariates in our analyses, selected based on previous research findings that indicated their relevance to secure messaging use or patient health outcomes. Not surprisingly given the differences we reported in the unadjusted changes in outcomes by patients’ control status, baseline values for all outcome measures were statistically associated with the change between baseline and endpoint. In our adjusted analyses, we did not find a statistical relationship with health insurance type, number of inpatient visits, number of message threads, rural home location, or differences between Whites and other races. We were unable to include ethnicity in our analyses due to limitations in the source data. Consistent with other research that has found disparities in health outcomes between Black and White patients [34,35], we observed that Black patients experienced increases in SBP when compared with White patients after controlling for all other covariates in the analysis. Given that some message content was associated with patient outcomes and that race is also associated with these outcomes, future studies should explore whether the message content patients send and receive is associated with their race.

A recent literature review found that the majority of studies detected no association between clinicians’ implicit bias and treatment recommendations when clinicians were asked to provide a diagnosis or treatment recommendation based on a written scenario [36]. Conversely, a different review noted that 5 of 6 observational and patient-reported measure–based studies found that physicians provided Black patients with less information than Whites [37]. Understanding how secure messaging communication varies by patient demographic characteristics and social determinants of health will be important to understanding how secure messaging might be used to improve patient outcomes in the future.

### Study Limitations

It is important to remember that this study examines the taxa in isolation; that is, a taxon is one component of the overall electronic conversation represented in each thread. From this research, we do not know what patient-generated messages preceded the staff response, so we cannot determine if, for example, *Orientation to procedures and treatments* responses answered patients’ questions or were appropriate responses to the patient-initiated request. Analyses that explore the call-and-response nature of the message thread—that consider the initiating request, final response, and the pathway to get to that final response—should yield more insight into these results. For example, patients who requested an appointment but received *Orientation to procedures and treatments* may have poorer outcomes than patients whose request was partially or completely fulfilled. It may also be that the number of clinic staff involved in responding to a thread, or the time taken to respond, has an impact on patient outcomes by increasing uncertainty or reducing patients’ trust [23]. Examining these factors might help explain why some of our findings do not align with our study hypotheses.

An important consideration for this research is that it demonstrates correlations and not causation. We hypothesized that *Self-reporting*, patients’ *Appreciation and praise* for clinic staff, and staff *Encouragement* would be associated with improved outcomes but we found the opposite: poorer DBP and A1C values in 2018 were associated with the *Encouragement* and *Appreciation and praise* taxa and patients who self-reported biometrics experienced increased SBP between the 2 years. Our outcomes were based on measurements obtained before and after the message collection period. If instead we obtained measurements in parallel to the secure messaging period, it is possible that we might have different results. For example, effects observed in 2018 may have less relevance to messages sent earlier in the calendar year (eg, patients only sent messages in the first quarter or half of the year). Another avenue of future study would include adding in more frequent measurements and exploring ways to identify any long-term impacts associated with certain taxa.

The Street et al [11] framework highlights intermediate outcomes on the pathway between communication functions and health outcomes. A proxy for the access to care construct, for example, might be overall health care utilization or whether the patients follow routine guidelines for care (eg, diabetic eye and foot examinations, or routine follow-up or preventive care appointments). Other constructs that could be measured with existing secondary data include self-care which might include the appropriate medication refill rates. These proximal outcomes also align to ones known to be associated with patient activation [38,39], further reinforcing the benefit to conducting these analyses. Future studies should explore associations between these taxa and those proximal and intermediate outcomes.

Responses to patients’ messages are typically triaged by clinic staff such that only the most complex messages are shared with physicians [40-42]. Effective workflow design may be critical to effective and efficient responses. These workflows, however, may be very clinic and physician specific. Our study did not incorporate this aspect into our analysis, because we did not have data on workflow practices utilized by different clinics. We also know that communication behaviors vary by patient and clinician characteristics [25-29,43-48]. Our study controlled for patient characteristics, but not clinician characteristics. Although we captured the type of clinic staff who responded to individual messages, we did not control for that in our analyses because we aggregated counts based on patient and patients typically had responses from a variety of clinic staff types. Future studies should conduct analyses at the clinician and clinic levels to account for workflow and communication practice differences.

Among our patient population, we found patients with controlled glycemic levels or blood pressure had a mean increase over the study period, whereas patients with uncontrolled glycemic or blood pressure had a mean decrease. Our study was not powered sufficiently to identify associations based on whether patients’ A1C or blood pressure was controlled at baseline [21]. It is likely, however, that patients’ communication and clinic staff responses differ based on patients’ current health status. We controlled for patients’ baseline values for each health outcome measure and these values were significantly associated with all the outcomes (*P*<.001), indicating a need for further research to better understand the differences in taxa use based on patients’ baseline health status.

We used only messages saved to patients’ charts. If clinic staff did not opt to save a message to the chart, it would not be captured in this study. We expect, therefore, that the numbers presented in this paper underestimate the number of messages sent and received by patients. We also expect that the number of nonresponses was underrepresented because it seems likely that if clinic staff did not respond to a message, they would be less likely to save the message as well. It is also possible that messages we classified as nonresponse had a response that was not saved to patients’ charts. If we assume that our sample underestimated the number of messages sent and received by patients, we would expect a bias toward the null and our results should therefore be viewed as conservative estimates of effect.

To our knowledge, only one 1 other study quantified clinic nonresponse to patients’ messages. Our study is the first to quantify nonresponse with a large number of messages and to link nonresponse to patient outcomes. The study by Lanham et al [49] study conducted chart reviews to determine if response occurred through other modalities and found that almost half of their 11 unanswered messages were resolved through other mechanisms. Our study did not assess responses by other communication modalities nor did it determine whether a response was warranted, but if we extrapolate the Lanham et al [49] findings to our work, that implies that only about half of the threads lacking a message response would have received a response not accounted for in our research (eg, phone, discussion during appointment). To better understand our study findings, it will be important to account for these other response types in future studies.

It is possible that thread initiation may be an indication of patient activation and engagement and clinic nonresponse may not inhibit patients’ activation. Patient activation follows 4 stages: belief in the importance of engagement in the care processes; knowledge in what is needed to improve health; taking action to improve or maintain health; and finally, maintaining or persisting in those actions even when stressed [31]. Patients at higher stages of activation generally experience better outcomes, have lower health care costs, and higher rates of health screening and prevention activities [32,38,39,50]. Alexander et al [51] found that patients who communicated outside of office visits had higher patient activation rates. Consistent with their research, we found that patients who initiated threads experienced A1C improvements compared to patients who did not initiate threads.

Several of our taxa had poor interrater reliability scores and as a result, these findings should be viewed with caution. It is notable that none of these taxa were statistically associated with our outcomes, perhaps due to the potential lack of clarity in their definitions. Future studies using this taxonomy should seek to clarify the definitions for these taxa.

### Conclusion

This is the first study to explore associations between message content and patient health outcomes. We identified associations between certain patient- and clinic staff–generated taxa and changes in patients’ glycemic levels and blood pressure. We also found that staff nonresponse was associated with improvements in patients’ DBP, although the reasoning behind this association is unclear. Significantly more research is needed to better understand what we observed in our study, including exploring the context of the full electronic conversation and outcomes, examining the temporal relationships between outcomes and message content, evaluating the impact of the potential confounder of patients’ activation, exploring other intermediate outcomes that might be better measures of effect, and incorporating other communication modalities to capture responses that occur outside of secure messaging. In the meantime, health care staff should be aware that message content is associated with patients’ health outcomes when corresponding with patients through this medium.

## Appendix

Multimedia Appendix 1 Secure message taxonomy.

Multimedia Appendix 2 Secure message taxa interrater and intrarater reliability.

Multimedia Appendix 3 Taxa associations with health outcomes.

## Abbreviations

DBP: diastolic blood pressure
SBP: systolic blood pressure
