# Supplementary material for: Associations Between Patient Health Outcomes and Secure Message Content Exchanged Between Patients and Clinicians: Retrospective Cohort Study
Source: J Med Internet Res. 2020 Oct 29;22(10):e19477. doi: 10.2196/19477 (PMC7661231; doi:10.2196/19477)
Supplement: Multimedia Appendix 1 [file jmir_v22i10e19477_app1.docx]

**When What You Say Matters: Associations Between Secure Message Content and Patient Health Outcomes**

**Multimedia Appendix 1. Secure Message Taxonomy**

| **Patient- or Clinician-Generated?** | **Level 1 Taxon** | **Level 2 Taxon** | **Level 3 Taxon** | **Definition** | **Examples** |
| --- | --- | --- | --- | --- | --- |
| Patient | Information seeking | Logistics | -- | Questions about timing, clinical processes, healthcare settings, or a patient’s care plan; questions for which a patient might reasonably expect most clinic staff to be able to provide an answer (does not necessarily require a physician’s response) | Questions about process and timing of medication or other treatment management (e.g., change in prescription, medication dosage); how to interpret laboratory results; why a test is being performed or a medication is necessary; how to prepare for the test or procedure upcoming diagnostic procedures; or what routine is needed for the medication |
| Patient |  | Medical guidance | -- | Questions that seek medical guidance or information; it is likely that the patient expects a physician or advanced medical training to provide a clinically based answer | Questions about the presence/absence of symptoms, symptom duration, symptom severity, symptom relevance specific to a health condition, including questions related to symptoms associated with side effects of medications, treatments, or procedures; treatment changes in context of symptoms/health condition (not process); generic questions about "is there something I can take for X symptom" |
| Patient | Information sharing | Clinical update | -- | Patient sharing information with clinician that does not require immediate action or a response (and may not require action at all); | Reporting results of clinical tests, procedures, or outcomes of visits with a different clinician or healthcare facility; |
| Patient |  | Response to clinician’s message | -- | Patient response to clinician’s question in preceding message within the thread. | Reporting symptoms/condition status in response to a clinical question, providing an update to clinician, or otherwise responding to clinician’s comment in preceding message |
| Patient |  | Self-reporting | -- | Patient sharing biometrics or other health-related self-measurements | Patient reports self-measured biomedical results (e.g., blood pressure, weight, glucose) not in response to a clinical question sent via secure messaging; food history |
| Patient | Task-oriented | Prescription refills and renewals requests | -- | Request for prescription refill or renewal | Request for medication or medical device prescription to be refilled or renewed |
| Patient |  | New or change prescription request | -- | Request for a new prescription or switch to a different medication | My medicine isn’t working, and I’d like to try a different insulin—will you please send the script to my pharmacy? |
| Patient |  | Other administrative | -- | Process-related requests that are administrative in nature | Requests for sick notes, contact information, medical records, patient portal access, or information about billing or insurance; technology-related questions related to interfacing with the patient portal or other patient-facing technology; requests for call or email |
| Patient |  | Referral requests | -- | Request for referral to other healthcare facility or clinician | I’d like to see a {specialist type}, would you enter a referral? |
| Patient |  | Scheduling request | Cancellation | Request to cancel existing appointment with no associated request to change the date or time. | Please cancel my appointment. |
| Patient |  |  | Follow-up | Request for an appointment relative to an existing health condition | I need to schedule my 3-month follow-up appointment with Dr. X. |
| Patient |  |  | Laboratory test or diagnostic procedure | Request for a laboratory test or diagnostic procedure (e.g., x-ray, ultrasound) order | I need my 3-month glucose check, would you enter the orders in the system? |
| Patient |  |  | New condition or symptom | Patient request for an appointment relative to a newly identified health condition or new symptom for existing condition; new patient appointment | I’ve had a cough and sore throat for 3 days now and would like to see the doctor. |
| Patient |  |  | Preventive care or physical exam | Request for a preventive care or routine exam | I’d like to schedule my annual physical exam. |
| Patient |  |  | Reschedule | Request for appointment to be changed to another date or time | I need to reschedule my upcoming appointment. |
| Clinician | Action responses | Acknowledge | -- | The response includes a recognition that the request for action or information is made, or that the message was received, but no indication is provided about whether the request will be fulfilled. | - Thank you for keeping us updated. - We received the picture. - Received. |
| Clinician |  | Denies | -- | The response indicates that the request will not be fulfilled | - The Outpatient Scheduling Center is unable to complete your request - We will not be able to come and give you a prescription any earlier than 12/28. - The doctor does not place orders for that. |
| Clinician |  | Fulfills request | -- | The response includes documentation that the request action was completed | - I rescheduled your appointment as noted below. - I sent the prescription to the pharmacy. - I have filled your form and attached here. |
| Clinician |  | Partially fulfills request | -- | The response indicates that there are additional steps that are necessary to fulfill the request, or that only part of the request can or has been completed | - I am working on getting him into Ortho really soon. Trying for this week. I will let you know. - Medication proposal sent to provider. - I will look at the schedule and figure out when we can see you! - I will resend it to the lab this afternoon. |
| Clinician | Information seeking | -- | -- | Clinic staffs’ requests for information | - Would you like to see another provider? - Have you had any difficulty with moderate sedation before? - Can you tell me what you are currently taking for your shortness of breath and wheezing at this time? - How are your home blood pressures? |
| Clinician | Information sharing | Deferred | -- | Clinical responses that refer the patient to another clinician for a response, or postpone an answer pending additional clinical information | - I will forward your message to {name}. - Ok. I will discuss with Dr {name}. - We can talk about transition of insulin when you see me. - A message has been forwarded to the clinic for review. - You will need to call their office to discuss a date to have the battery replaced in your stimulator. - We do not have the answer yet. |
| Clinician |  | Medical guidance | -- | Answer requires medical training/ provision of clinical information; requires medical decision-making | Clinician provides treatment decisions, gives care instructions, dietary guidance, instructs the patient on the best next steps in his or her care plan, interprets diagnostic procedure or laboratory results, or provides information on symptoms or the patient’s health condition. |
| Clinician |  | Orientation to procedures, treatments, or preventive behaviors | -- | Process answers; responses explain what a patient might expect during a treatment or diagnostic procedure, or in a new healthcare setting or situation | - You can call [phone number] to schedule that appointment. - The compression stockings haven't been mailed yet, because it turns out that Medicare will only cover them if we order a higher level of compression (30-40 mmHg). - I just checked our inbox and I don't see any recent lab results. - Every portal message sent to a provider on our team comes to a central message pool which is monitored by 2 Triage nurses. - I just called MRI sched and was advised that you should arrive 45 min before the 1st appt-but should not be a problem. The 3rd appt is with Dr {name} in Neurology. |
| Clinician | Task-oriented | Recommendation to schedule an appointment | -- | Clinician suggests that patient schedule an appointment | - In light of this, I WOULD make a follow-up appt with him. - Can you come in on Friday morning (as early as you can, maybe 8:00) and we will see how your labs look and see if we can figure out what’s going on. - Since I am refilling your medications, I will need to see you 6 months from 2/2017 which would be 8/2017. - I would like you to have follow-up labs early next week. |
| Both | Social communication | Appreciation and praise | -- | Content that expresses gratitude or offers acknowledgement or appreciation of a service provided, health status, or another act | - Thanks for any magic you can work! - I really appreciate your understanding how much it helps give me some peace of mind. - I wanted to take the opportunity to thank you for allowing me to continue with the Housecalls program. This program provided me with outstanding patient care above and beyond the typical call of duty. - What a GREAT program. I'm loving this! You guys rock and have made such a difference in my life |
| Patient |  | Complaints | -- | Expressions of frustration or displeasure about service or life issues | - I have asked and asked and asked as to when the podiatrist is coming back and they will not or cannot give me an answer. All I can get out of them are that the podiatrist only comes once a month. - The encounter with this current provider has proved to be not beneficial to my health. - I don't feel like I should be treated like a child when it comes to my pain medicine. - {doctor’s name} is a jerk. |
| Patient |  | Life issues | -- | Communication about aspects of the patients’ life not specifically related to health | - First of all I want to apologize for my wife for the way that I let my temper get to me when I spoke with {name} a few moments ago. I am very sorry for my behavior. - Trying to figure out what caused my munchies today. I was up at 2am and couldn't get back to sleep and was stressed a bit - I also wanted to tell you I was 're-introduced' to a girl I went to high school with who has POTS and EDS, and a pacemaker. I didn't know until today that she is one of your patients. Scary how similar parts of our stories are. - Shared funny comic with note “Here is another, perhaps more subtle, response for many situations.” - Since I've seen you, there was a fire in the apartment next to mine, the sprinklers there went off, and my apartment got flooded. The contractors hired to repair the damage made a complete mess of my place. |
| Clinician |  | Encouragement | -- | An expression of positive reinforcement or good feelings of the provider in regard to patient's actions, possessions, or self | - Blood pressures are great. - It sounds like you are really trying hard to quit and you should be proud of yourself. Please keep up the good work and will see you at your next appointment - Your blood sugar is NORMAL OFF INSULIN! Is that amazing or what. You are a rock star. - Looking better and better!! Great job! |
