# Supplementary material for: Associations Between Patient Health Outcomes and Secure Message Content Exchanged Between Patients and Clinicians: Retrospective Cohort Study
Source: J Med Internet Res. 2020 Oct 29;22(10):e19477. doi: 10.2196/19477 (PMC7661231; doi:10.2196/19477)
Supplement: Multimedia Appendix 2 [file jmir_v22i10e19477_app2.docx]

**When What You Say Matters: Associations Between Secure Message Content and Patient Health Outcomes**

**Multimedia Appendix 2. Secure Message Taxa Interrater and Intrarater Reliability**

| Taxa | Inter-rater reliability | | Intra-rater reliability | |
| --- | --- | --- | --- | --- |
|  | Final round Kappa estimates [95% CI] | Reliability interpretation | Kappa estimates [95% CI] | Reliability interpretation |
| Patient-and clinician-generated Social communication |  |  |  |  |
| --summary-- | 0.55 [0.29 ,0.81] | Fair | 0.67 [0.57, 0.77] | Good |
| Appreciation/praise | 0.57 [0.28, 0.86] | Fair | 0.79 [0.67, 0.91] | Excellent |
| Complaints | N/A | N/A | 0.72 [0.52, 0.92] | Good |
| Life issues | 0.50 [-0.10, 1.00] | Fair | 0.40 [0.17, 0.62] | Fair |
| Clinician-generated |  |  |  |  |
| Action responses |  |  |  |  |
| Fulfilled request | 0.74 [0.56, 0.92] | Good | 0.85 [0.80, 0.89] | Excellent |
| Acknowledge | 0.58 [0.33, 0.84] | Fair | 0.75 [0.66, 0.84] | Excellent |
| Partially fulfill request | 0.49 [0.14, 0.83] | Fair | 0.54 [0.42, 0.66] | Fair |
| Denies | -0.01 [-0.02, 0.00] | Poor | 0.43 [0.18, 0.69] | Fair |
| Information seeking | 0.85 [0.75, 0.96] | Excellent | 0.88 [0.85, 0.92] | Excellent |
| Information sharing |  |  |  |  |
| --summary-- | 0.75 [0.65 ,0.85] | Excellent | 0.77 [0.73, 0.80] | Excellent |
| Medical guidance | 0.86 [0.76, 0.95] | Excellent | 0.83 [0.79, 0.87] | Excellent |
| Orientation | 0.47 [0.30, 0.65] | Fair | 0.63 [0.58, 0.67] | Good |
| Deferred information sharing | 0.52 [0.20, 0.83] | Fair | 0.68 [0.61, 0.74] | Good |
| Recommendation to schedule | -0.01 [-0.03, 0.00] | Poor | 0.69 [0.54, 0.85] | Good |
| Patient-generated |  |  |  |  |
| Information seeking |  |  |  |  |
| --summary-- | 0.72 [0.59 ,0.85] | Good | 0.81 [0.77, 0.85] | Excellent |
| Medical guidance | 0.67 [0.51, 0.83] | Good | 0.81 [0.76, 0.86] | Excellent |
| Logistics | 0.29 [0.04, 0.54] | Poor | 0.69 [0.62, 0.75] | Good |
| Information sharing |  |  |  |  |
| --summary-- | 0.71 [0.59 ,0.82] | Good | 0.86 [0.83, 0.88] | Excellent |
| Self-reporting | 1.00 [1.00, 1.00] | Excellent | 0.89 [0.82, 0.95] | Excellent |
| Response to clinician | 0.51 [0.33, 0.70] | Fair | 0.85 [0.82, 0.89] | Excellent |
| Clinical update | 0.57 [0.36, 0.78] | Fair | 0.68 [0.62, 0.74] | Good |
| Prescription request |  |  |  |  |
| --summary-- | 0.83 [0.68 ,0.97] | Excellent | 0.89 [0.85, 0.93] | Excellent |
| Prescription refill/renewal | 0.82 [0.61, 1.00] | Excellent | 0.88 [0.82, 0.93] | Excellent |
| New or changed Rx | 0.56 [0.24, 0.87] | Fair | 0.69 [0.58, 0.80] | Good |
| Scheduling request |  |  |  |  |
| --summary-- | 0.45 [0.15 ,0.75] | Fair | 0.90 [0.86, 0.93] | Excellent |
| Cancellation | N/A | N/A | 0.95 [0.89, 1.00] | Excellent |
| Reschedule | N/A | N/A | 0.90 [0.84, 0.95] | Excellent |
| New condition/ symptom | 0.66 [0.05, 1.00] | Good | 0.86 [0.76, 0.96] | Excellent |
| Preventive care | N/A | N/A | 0.67 [0.39, 0.94] | Good |
| Follow-up appointment | 0.49 [0.06, 0.92] | N/A | 0.61 [0.45, 0.77] | Good |
| Lab or other diagnostic procedure | 0.40 [-0.15, 0.94] | Fair | 0.60 [0.40, 0.80] | Good |
| Other task-oriented request |  |  |  |  |
| Referral | 1.00 [1.00, 1.00] | Excellent | 0.78 [0.64, 0.92] | Excellent |
| Other administrative | 0.48  [0.17, 0.79] | Fair | 0.72 [0.62, 0.81] | Good |
