# Supplementary material for: Associations Between Patient Health Outcomes and Secure Message Content Exchanged Between Patients and Clinicians: Retrospective Cohort Study
Source: J Med Internet Res. 2020 Oct 29;22(10):e19477. doi: 10.2196/19477 (PMC7661231; doi:10.2196/19477)
Supplement: Multimedia Appendix 3 [file jmir_v22i10e19477_app3.docx]

Table 3-1. Linear Regression Results Estimating Associations Between Taxa and Glycemic Changes, Among the Secure Messaging Population

| **Level 1 Taxon** | **Level 2 Taxon** | **Level 3 Taxon** | **F-value** (all significant at *P*<.001) | **R-square** | **Taxon b weight (*P-*value)** | **b weight (*P*-value)** | | | | | | | | | | | | | |
| --- | --- | --- | --- | --- | --- | --- | --- | --- | --- | --- | --- | --- | --- | --- | --- | --- | --- | --- | --- |
|  |  |  |  |  |  | **Age (years)** | **Average distance to clinic** | **Male vs female** | **Other payer vs Private payer** | **Public payer vs Private payer** | **Uninsured vs Private payer** | **Black vs white** | **Other race vs white** | **Rural vs urban home location** | **Diabetes only vs Diabetes & hypertension** | **Baseline A1C (%)** | **Number of outpatient visits** | **Number of inpatient visits** | **Number of threads** |
| Information seeking | Grouped | -- | 13.48 | .43 | -.07 (.04) | -- | -- | -- | -- | -- | -- | -- | -- | -- | -- | -.54 (<.001) | -- | -- | -- |
|  | Logistics | -- | 13.42 | .43 | -- | -- | -- | -- | -- | -- | -- | -- | -- | -- | -- | -.54 (<.001) | -- | -- | -- |
|  | Medical guidance | -- | 13.09 | .42 | -- | -- | -- | -- | -- | -- | -- | -- | -- | -- | -- | -.54 (<.001) | -- | -- | -- |
| Information sharing | Grouped | -- | 13.59 | .43 | .08 (.02) | -- | -- | -- | -- | -- | -- | -- | -- | -- | -- | -.54 (<.001) | -- | -- | -- |
|  | Clinical update | -- | 13.16 | .43 | -- | -- | -- | -- | -- | -- | -- | -- | -- | -- | -- | -.54 (<.001) | -- | -- | -- |
|  | Response to clinician’s message | -- | 13.30 | .43 | -- | -- | -- | -- | -- | -- | -- | -- | -- | -- | -- | -.54 (<.001) | -- | -- | -- |
|  | Self-reporting | -- | 13.14 | .42 | -- | -- | -- | -- | -- | -- | -- | -- | -- | -- | -- | -.54 (<.001) | -- | -- | -- |
| Task-oriented | Prescription refills and renewals requests | -- | 13.02 | .42 | -- | -- | -- | -- | -- | -- | -- | -- | -- | -- | -- | .04 (<.001) | -- | -- | -- |
|  | New or change prescription request | -- | 13.00 | .42 | -- | -- | -- | -- | -- | -- | -- | -- | -- | -- | -- | -.54 (<.001) | -- | -- | -- |
|  | Other administrative | -- | 13.00 | .42 | -- | -- | -- | -- | -- | -- | -- | -- | -- | -- | -- | -.54 (<.001) | -- | -- | -- |
|  | Referral requests | -- | 12.99 | .42 | -- | -- | -- | -- | -- | -- | -- | -- | -- | -- | -- | <.001 | -- | -- | -- |
|  | Scheduling request | Cancellation | 13.04 | .42 | -- | -- | -- | -- | -- | -- | -- | -- | -- | -- | -- | -.54 (<.001) | -- | -- | -- |
|  |  | Follow-up | 13.01 | .42 | -- | -- | -- | -- | -- | -- | -- | -- | -- | -- | -- | -.54 (<.001) | -- | -- | -- |
|  |  | Laboratory test or diagnostic procedure | 13.04 | .42 | -- | -- | -- | -- | -- | -- | -- | -- | -- | -- | -- | -.54 (<.001) | -- | -- | -- |
|  |  | New condition or symptom | 12.99 | .42 | -- | -- | -- | -- | -- | -- | -- | -- | -- | -- | -- | -.54 (<.001) | -- | -- | -- |
|  |  | Preventive care or physical exam | 13.11 | .42 | -- | -- | -- | -- | -- | -- | -- | -- | -- | -- | -- | -.54 (<.001) | -- | -- | -- |
|  |  | Reschedule | 13.00 | .42 | -- | -- | -- | -- | -- | -- | -- | -- | -- | -- | -- | -.54 (<.001) | -- | -- | -- |
| Social communication | Grouped | -- | 13.26 | .43 | -- | -- | -- | -- | -- | -- | -- | -- | -- | -- | -- | -.54 (<.001) | -- | -- | -- |
|  | Appreciation and praise | -- | 13.04 | .42 | -- | -- | -- | -- | -- | -- | -- | -- | -- | -- | -- | -.54 (<.001) | -- | -- | -- |
|  | Complaints | -- | 13.15 | .42 | -- | -- | -- | -- | -- | -- | -- | -- | -- | -- | -- | -.54 (<.001) | -- | -- | -- |
|  | Life issues | -- | 13.10 | .42 | -- | -- | -- | -- | -- | -- | -- | -- | -- | -- | -- | -.54 (<.001) | -- | -- | -- |
| Action responses | Grouped | -- | 13.11 | .42 | -- | -- | -- | -- | -- | -- | -- | -- | -- | -- | -- | -.54 (<.001) | -- | -- | -- |
|  | Acknowledge | -- | 13.01 | .42 | -- | -- | -- | -- | -- | -- | -- | -- | -- | -- | -- | -.54 (<.001) | -- | -- | -- |
|  | Fulfills request | -- | 13.05 | .42 | -- | -- | -- | -- | -- | -- | -- | -- | -- | -- | -- | -.53 (<.001) | -- | -- | -- |
|  | Partially fulfills request | -- | 12.99 | .42 | -- | -- | -- | -- | -- | -- | -- | -- | -- | -- | -- | -.54 (<.001) | -- | -- | -- |
| Information seeking | -- | -- | 13.15 | .43 | -- | -- | -- | -- | -- | -- | -- | -- | -- | -- | -- | -.54 (<.001) | -- | -- | -- |
| Information sharing | Grouped | -- | 13.02 | .42 | -- | -- | -- | -- | -- | -- | -- | -- | -- | -- | -- | -.54 (<.001) | -- | -- | -- |
|  | Deferred | -- | 13.44 | .42 | -- | -- | -- | -- | -- | -- | -- | -- | -- | -- | -- | -.54 (<.001) | -- | -- | -- |
|  | Medical guidance | -- | 13.52 | .43 | .08 (.03) | -- | -- | -- | -- | -- | -- | -- | -- | -- | -- | -.54 (<.001) | -- | -- | -- |
|  | Orientation to procedures or treatments | -- | 13.24 | .43 | -- | -- | -- | -- | -- | -- | -- | -- | -- | -- | -- | -.54 (<.001) | -- | -- | -- |
| Social communication | Encouragement | -- | 13.73 | .44 | .51 (.01) | -- | -- | -- | -- | -- | -- | -- | -- | -- | -- | -.53 (<.001) | -- | -- | -- |
| Non-response* | -- | -- | 13.35 | .43 | -- | -- | -- | -- | -- | -- | -- | -- | -- | -- | -- | -.54 (<.001) | -- | -- | -- |

Notes: N=607. Includes only patients who initiated at least one secure message thread in 2017. Each row represents a linear regression model where the dependent variable is the change in A1C percentage between 2016 and 2018. The independent variable is the taxon prevalence and the covariates are the patient characteristics. -- Not significant at *P*<.05. *Non-response is not a taxon; rather, it is identified as any message thread that includes no clinic staff secure message response.

Table 3-2. Linear Regression Results Estimating Associations Between Taxa and Glycemic Changes, Among the Full Population

| **Level 1 Taxon** | **Level 2 Taxon** | **Level 3 Taxon** | **N** | **F-value** (all significant at *P*<.001) | **R-square** | **Taxon b weight (*P*-value)** | **b weight (*P*-value)** | | | | | | | | | | | | |
| --- | --- | --- | --- | --- | --- | --- | --- | --- | --- | --- | --- | --- | --- | --- | --- | --- | --- | --- | --- |
|  |  |  |  |  |  |  | **Age (years)** | **Male vs female** | **Other payer vs Private payer** | **Public payer vs Private payer** | **Uninsured vs Private payer** | **Black vs white** | **Other race vs white** | **Rural vs urban home location** | **Diabetes only vs Diabetes & hypertension** | **Baseline A1C (%)** | **Number of outpatient visits** | **Number of inpatient visits** | **Number of threads** |
| Information seeking | Grouped | -- | 433 | 11.79 | .28 | -.08 (.01) | -- | -- | -- | -- | -- | -- | -- | -- | -- | -.42 (<.001) | -- | -- | -- |
|  | Logistics | -- | 369 | 1.98 | .30 | -.11 (.04) | -- | -- | -- | -- | -- | -- | -- | -- | -- | -.44 (<.001) | -- | -- | -- |
|  | Medical guidance | -- | 395 | 8.18 | .23 | -- | -- | -- | -- | -- | -- | -- | -- | -- | -- | -.36 (<.001) | -- | -- | -.02 (.03) |
| Information sharing | Grouped | -- | 438 | 11.45 | .27 | -- | -- | -- | -- | -- | -- | -- | -- | -- | -- | -.40 (<.001) | -- | -- | -.02 (.03) |
|  | Clinical update | -- | 380 | 8.74 | .25 | -- | -- | -- | -- | -- | -- | -- | -- | -- | -- | -.38 (<.001) | -- | -- | -- |
|  | Response to clinician’s message | -- | 390 | 1.70 | .29 | -- | -- | -- | -- | -- | -- | -- | -- | -- | -- | -.40 (<.001) | -- | -- | -.02 (.04) |
|  | Self-reporting | -- | 284 | 6.15 | .24 | -- | -- | -- | -- | -- | -- | -- | -- | -- | -- | -.35 (<.001) | -- | -- | -.02 (<.01) |
| Task-oriented | Prescription refills and renewals requests | -- | 385 | 9.14 | .26 | -- | -- | -- | -- | -- | -- | -- | -- | -- | -- | -.37 (<.001) | -- | -- | -.02 (.02) |
|  | New or change prescription request | -- | 340 | 7.59 | .25 | -- | -- | -- | -- | -- | -- | -- | -- | -- | -- | -.34 (<.001) | -- | -- | -.02 (.01) |
|  | Other administrative | -- | 332 | 7.35 | .25 | -- | -- | -- | -- | -- | -- | -- | -- | -- | -- | -.35 (<.001) | -- | -- | -.02 (.04) |
|  | Referral requests | -- | 277 | 5.68 | .23 | -- | -- | -- | -- | -- | -- | -- | -- | -- | -- | -.36 (<.001) | -- | -- | -.03 (.02) |
|  | Scheduling request | Cancellation | 305 | 4.96 | .19 | -- | -- | -- | -- | -- | -- | -- | -- | -- | -- | -.32 (<.001) | -- | -- | -- |
|  |  | Follow-up | 318 | 5.76 | .21 | -- | -- | -- | -- | -- | -- | -- | -- | -- | -- | -.32 (<.001) | -- | -- | -- |
|  |  | Laboratory test or diagnostic procedure | 294 | 6.08 | .23 | -- | -- | -- | -- | -- | -- | -- | -- | -- | -- | -.37 (<.001) | -- | -- | -- |
|  |  | New condition or symptom | 291 | 5.34 | .21 | -- | -- | -- | -- | -- | -- | -- | -- | -- | -- | -.32 (<.001) | -- | -- | -.02 (.04) |
|  |  | Preventive care or physical exam | 266 | 4.35 | .20 | -- | -- | -- | -- | -- | -- | -- | -- | -- | -- | -.30 (<.001) | -- | -- | -.04 (.04) |
|  |  | Reschedule | 338 | 8.31 | .26 | -- | -- | -- | -- | -- | -- | -- | -- | -- | -- | -.36 (<.001) | -- | -- | -.02 (.01) |
| Social communication | Grouped | -- | 306 | 7.22 | .26 | -- | -- | -- | -- | -- | -- | -- | -- | -- | -- | -.37 (<.001) | -- | -- | -- |
|  | Appreciation and praise | -- | 265 | 4.58 | .20 | -- | -- | -- | -- | -- | -- | -- | -- | -- | -- | -.32 (<.001) | -- | -- | -- |
|  | Complaints | -- | 268 | 5.92 | .25 | -- | -- | -- | -- | -- | -- | -- | -- | -- | -- | -.37 (<.001) | -- | -- | -- |
|  | Life issues | -- | 273 | 4.11 | .18 | -- | -- | -- | -- | -- | -- | -- | -- | -- | -- | -.30 (<.001) | -- | -- | -- |
| Action responses | Grouped | -- | 460 | 12.79 | .29 | -- | -- | -- | -- | -- | -- | -- | -- | -- | -- | -.40 (<.001) | -- | -- | -.02 (.01) |
|  | Acknowledge | -- | 316 | 5.93 | .22 | -- | -- | -- | -- | -- | -- | -- | -- | -- | -- | -.33 (<.001) | -- | -- | -- |
|  | Fulfills request | -- | 443 | 12.44 | .29 | -- | -- | -- | -- | -- | -- | -- | -- | -- | -- | -.40 (<.001) | -- | -- | -.02 (.01) |
|  | Partially fulfills request | -- | 317 | 7.29 | .25 | -- | -- | -- | -- | -- | -- | -- | -- | -- | -- | -.35 (<.001) | -- | -- | -- |
| Information seeking | -- | -- | 407 | 13.19 | .32 | -- | -- | -- | -- | -- | -- | -- | -- | -- | -- | -.44 (<.001) | -- | -- | -.02 (.02) |
| Information sharing | Grouped | -- | 483 | 13.08 | .28 | -- | -- | -- | -- | -- | -- | -- | -- | -- | -- | -.40 (<.001) | -- | -- | -- |
|  | Deferred | -- | 379 | 9.28 | .26 | -- | -- | -- | -- | -- | -- | -- | -- | -- | -- | -.36 (<.001) | -- | -- | -.02 (.02) |
|  | Medical guidance | -- | 388 | 8.71 | .25 | -- | -- | -- | -- | -- | -- | -- | -- | -- | -- | -.37 (<.001) | -- | -- | -.02 (.02) |
|  | Orientation to procedures or treatments | -- | 449 | 12.87 | .29 | -.07 (.02) | -- | -- | -- | -- | -- | -- | -- | -- | -- | -.41 (<.001) | -- | -- | -- |
| Social communication | Encouragement | -- | 266 | 4.76 | .21 | .16 (.02) | -- | -- | -- | -- | -- | -- | -- | -- | -- | -.31 (<.001) | -- | -- | -- |
| Non-response* | -- | -- | 430 | 12.80 | .30 | -- | -- | -- | -- | -- | -- | -- | -- | -- | -- | -.42 (<.001) | -- | -- | -- |

Notes: Includes patients sent or received at least one message coded with the selected taxon plus all patients who did not initiate a thread in 2017. Each row represents a linear regression model where the dependent variable is the change in A1C percentage between 2016 and 2018. The independent variable is the taxon prevalence and the covariates are the patient characteristics. -- Not significant at *P*<.05. *Non-response is not a taxon, rather, it is identified as any message thread that includes no clinic staff secure message response.

Table 3-3. Linear Regression Results Estimating Associations Between Taxa and Systolic Blood Pressure Changes, Among the Secure Messaging Population

| **Level 1 Taxon** | **Level 2 Taxon** | **Level 3 Taxon** | **F-value** (all significant at *P*<.001) | **R-square** | **Taxon b weight  (*P*-value)** | **b weight (*P*-value)** | | | | | | | | | | | | | | |
| --- | --- | --- | --- | --- | --- | --- | --- | --- | --- | --- | --- | --- | --- | --- | --- | --- | --- | --- | --- | --- |
|  |  |  |  |  |  | **Age (years)** | **Average distance to clinic** | **Male vs female** | **Other payer vs Private payer** | **Public payer vs Private payer** | **Uninsured vs Private payer** | **Black vs white** | **Other race vs white** | **Rural vs urban home location** | **Hypertension only vs Diabetes & hypertension** | **Baseline DBP** | **Baseline SBP** | **Number of outpatient visits** | **Number of inpatient visits** | **Number of threads** |
| Information seeking | Grouped |  | 11.85 | .24 | -- | .15 (.04) | -- | -- | -- | -- | -- | 4.07 (.02) | -- | -- | -- | -- | -.58 (<.001) | -- | -- | -- |
|  | Logistics | -- | 11.82 | .24 | -- | .14 (.04) | -- | -- | -- | -- | -- | 4.08 (.02) | -- | -- | -- | -- | -.58 (<.001) | -- | -- | -- |
|  | Medical guidance | -- | 11.84 | .24 | -- | .15 (.04) | -- | -- | -- | -- | -- | 4.13 (.02) | -- | -- | -- | -- | -.58 (<.001) | -- | -- | -- |
| Information sharing | Grouped |  | 11.86 | .24 | -- | -- | -- | -- | -- | -- | -- | 4.22 (.02) | -- | -- | -- | -- | -.58 (<.001) | -- | -- | -- |
|  | Clinical update | -- | 11.80 | .24 | -- | -- | -- | -- | -- | -- | -- | 4.17 (.02) | -- | -- | -- | -- | -.58 (<.001) | -- | -- | -- |
|  | Response to clinician’s message | -- | 11.82 | .24 | -- | .14 (.04) | -- | -- | -- | -- | -- | 4.12 (.02) | -- | -- | -- | -- | -.58  (<.001) | -- | -- | -- |
|  | Self-reporting | -- | 12.20 | .25 | 1.72 (.03) | -- | -- | -- | -- | -- | -- | 4.02 (.02) | -- | -- | -- | -- | -.59 (<.001) | -- | -- | -- |
| Task-oriented | Prescription refills and renewals requests | -- | 12.10 | .25 | -- | .16 (.03) | -- | -- | -- | -- | -- | 4.38 (.02) | -- | -- | -- | -- | -.59 (<.001) | -- | -- | -- |
|  | New or change prescription request | -- | 11.83 | .24 | -- | .14 (.04) | -- | -- | -- | -- | -- | 4.12 (.02) | -- | -- | -- | -- | -.58 (<.001) | -- | -- | -- |
|  | Other administrative | -- | 11.81 | .24 | -- | .15 (.04) | -- | -- | -- | -- | -- | 4.16 (.02) | -- | -- | -- | -- | -.58 (<.001) | -- | -- | -- |
|  | Referral requests | -- | 11.87 | .24 | -- | .15 (.04) | -- | -- | -- | -- | -- | 4.22 (.02) | -- | -- | -- | -- | -.58 (<.001) | -- | -- | -- |
|  | Scheduling request | Cancellation | 11.82 | .24 | -- | -- | -- | -- | -- | -- | -- | 4.07(.02) | -- | -- | -- | -- | -.58 (<.001) | -- | -- | -- |
|  |  | Follow-up | 11.83 | .24 | -- | .15 (.04) | -- | -- | -- | -- | -- | 4.16 (.02) | -- | -- | -- | -- | -.58 (<.001) | -- | -- | -- |
|  |  | Laboratory test or diagnostic procedure | 11.83 | .24 | -- | .14 (.04) | -- | -- | -- | -- | -- | 4.11 (.02) | -- | -- | -- | -- | -.58 (<.001) | -- | -- | -- |
|  |  | New condition or symptom | 11.90 | .24 | -- | .16 (.03) | -- | -- | -- | -- | -- | 4.20 (.02) | -- | -- | -- | -- | -.58 (<.001) | -- | -- | -- |
|  |  | Preventive care or physical exam | 11.81 | .24 | -- | .15 (.04) | -- | -- | -- | -- | -- | 4.16 (.02) | -- | -- | -- | -- | -.58 (<.001) | -- | -- | -- |
|  |  | Reschedule | 12.01 | .24 | -- | .16 (.03) | -- | -- | -- | -- | -- | 3.82 (.03) | -- | -- | -- | -- | -.58 (<.001) | -- | -- | -- |
| Social communication | Grouped | -- | 11.80 | .24 | -- | .14 (.04) | -- | -- | -- | -- | -- | 4.14 (.02) | -- | -- | -- | -- | -.58 (<.001) | -- | -- | -- |
|  | Appreciation and praise | -- | 12.22 | .25 | 5.80 (.02) | -- | -- | -- | -- | -- | -- | 4.08 (.02) | -- | -- | -- | -- | -.58 (<.001) | -- | -- | -- |
|  | Complaints | -- | 12.04 | .25 | -- | -- | -- | -- | -- | -- | -- | 4.04 (.02) | -- | -- | -- | -- | -.58 (<.001) | -- | -- | -- |
|  | Life issues | -- | 11.81 | .24 | -- | .14 (.04) | -- | -- | -- | -- | -- | 4.12 (.02) | -- | -- | -- | -- | -.58 (<.001) | -- | -- | -- |
| Action responses | Grouped | -- | 11.99 | .24 | -- | -- | -- | -- | -- | -- | -- | 4.15 (.02) | -- | -- | -- | -- | -0.58 (<.001) | -- | -- | -- |
|  | Acknowledge | -- | 11.80 | .24 | -- | .14 (.04) | -- | -- | -- | -- | -- | 4.14 (.02) | -- | -- | -- | -- | -0.58 (<.001) | -- | -- | -- |
|  | Fulfills request | -- | 11.92 | .24 | -- | -- | -- | -- | -- | -- | -- | 4.12 (.02) | -- | -- | -- | -- | -.58 (<.001) | -- | -- | -- |
|  | Partially fulfills request | -- | 11.88 | .24 | -- | .15 (.04) | -- | -- | -- | -- | -- | 4.30 (.02) | -- | -- | -- | -- | -.58 (<.001) | -- | -- | -- |
| Information seeking | -- | -- | 11.82 | .24 | -- | .14 (.04) | -- | -- | -- | -- | -- | 4.10 (.02) | -- | -- | -- | -- | -.58 (<.001) | -- | -- | -- |
| Information sharing | Grouped | -- | 11.85 | .24 | -- | -- | -- | -- | -- | -- | -- | 4.17 (.02) | -- | -- | -- | -- | -.58 (<.001) | -- | -- | -- |
|  | Deferred | -- | 12.46 | .25 | 1.28 (<.01) | .15 (.03) | -- | -- | -- | -- | -- | 4.32 (.02) | -- | -- | -- | -- | -.58 (<.001) | -- | -- | -- |
|  | Medical guidance | -- | 11.84 | .24 | -- | .15 (.04) | -- | -- | -- | -- | -- | 4.17 (.02) | -- | -- | -- | -- | -.58 (<.001) | -- | -- | -- |
|  | Orientation to procedures or treatments | -- | 11.83 | .24 | -- | .14 (.04) | -- | -- | -- | -- | -- | 4.18 (.02) | -- | -- | -- | -- | -.58 (<.001) | -- | -- | -- |
| Social communication | Encouragement | -- | 11.87 | .24 | -- | .15 (.04) | -- | -- | -- | -- | -- | 4.15 (.02) | -- | -- | -- | -- | -.58 (<.001) | -- | -- | -- |
| Non-response* | -- | -- | 11.98 | .24 | -- | .15 (.03) | -- | -- | -- | -- | -- | 4.04 (.02) | -- | -- | -- | -- | -.58 (<.001) | -- | -- | -- |

Notes: N=607. DBP=Diastolic blood pressure; SBP=Systolic blood pressure. Includes only patients who initiated at least one secure message thread in 2017. Each row represents a linear regression model where the dependent variable is the change in SBP percentage between 2016 and 2018. The independent variable is the taxon prevalence and the covariates are the patient characteristics. -- Not significant at *P*<.05. *Non-response is not a taxon; rather, it is identified as any message thread that includes no clinic staff secure message response.

Table 3-4. Linear Regression Results Estimating Associations Between Taxa and Systolic Blood Pressure Changes, Among the Full Population

| **Level 1 Taxon** | **Level 2 Taxon** | **Level 3 Taxon** | **N** | **F-value** (all significant at *P*<.001) | **R-square** | **Taxon b weight (*P*-value)** | **b weight (*P*-value)** | | | | | | | | | | | | | |
| --- | --- | --- | --- | --- | --- | --- | --- | --- | --- | --- | --- | --- | --- | --- | --- | --- | --- | --- | --- | --- |
|  |  |  |  |  |  |  | **Age (years)** | **Male vs female** | **Other payer vs Private payer** | **Public payer vs Private payer** | **Uninsured vs Private payer** | **Black vs white** | **Other race vs white** | **Rural vs urban home location** | **HTN vs Both conditions** | **Baseline DBP** | **Baseline SBP** | **Number of OVs** | **Number of inpatient visits** | **Number of threads** |
| Information seeking | Grouped | -- | 1022 | 25.36 | .27 | -- | -- | -- | -- | -- | -- | 3.64 (<.01) | -- | -- | -3.50 (<.01) | -- | -.60 (<.001) | -- | -- | -- |
|  | Logistics | -- | 885 | 22.73 | .28 | -- | -- | -- | -- | -- | -- | 4.26 (<.01) | -- | -- | -4.47 (<.001) | -- | -.61 (<.001) | -- | -- | -- |
|  | Medical guidance | -- | 965 | 23.14 | .27 | -- | -- | -- | -- | -- | -- | 3.77 (<.01) | -- | -- | -3.53 (.01) | -- | -.59 (<.001) | -- | -- | -- |
| Information sharing | Grouped | -- | 1034 | 23.86 | .26 | -- | -- | -- | -- | -- | -- | 4.37 (<.001) | -- | -- | -3.22 (<.01) | -- | -.59 (<.001) | -- | -- | -- |
|  | Clinical update | -- | 909 | 22.02 | .27 | -- | -- | -- | -- | -- | -- | 3.41 (.01) | -- | -- | -3.04 (.02) | -- | -.57 (<.001) | -- | -- | -- |
|  | Response to clinician’s message | -- | 937 | 23.53 | .28 | -- | -- | -- | -- | -- | -- | 4.15 (<.01) | -- | -- | -3.76 (<.01) | -- | -.60 (<.001) | -- | -- | -- |
|  | Self-reporting | -- | 684 | 16.35 | .27 | 1.60 (.04) | -- | -- | -- | -- | -- | 4.23 (.01) | -- | -- | -3.84 (.01) | -- | -.55 (<.001) | -- | -- | -- |
| Task-oriented | Prescription refills and renewals requests | -- | 925 | 22.69 | .27 | -- | -- | -- | -- | -- | -- | 4.04 (<.01) | -- | -- | -3.61 (<.01) | -- | -.61 (<.001) | -- | -- | -- |
|  | New or change prescription request | -- | 784 | 2.27 | .28 | -- | -- | -- | -- | -- | -- | 3.74 (.01) | -6.34 (0.04) | -- | -3.67 (<.01) | -- | -.59 (<.001) | -- | -- | -- |
|  | Other administrative | -- | 806 | 22.35 | .30 | -- | -- | -- | -- | -- | -- | 3.45 (.02) | -- | -- | -4.44 (<.001) | -- | -.59 (<.001) | -- | -- | -- |
|  | Referral requests | -- | 692 | 18.10 | .29 | -- | -- | -- | -- | -- | -- | 3.39 (.03) | -- | -- | -4.60 (<.01) | -- | -.62 (<.001) | -- | -- | -- |
|  | Scheduling request | Cancellation | 757 | 21.22 | .30 | -- | -- | -- | -- | -- | -- | 4.42 (<.01) | -- | -- | -5.02 (<.001) | -- | -.59 (<.001) | -- | -- | -- |
|  |  | Follow-up | 764 | 18.76 | .27 | -- | -- | -- | -- | -- | -- | 3.48 (.02) | -- | -- | -4.51 (<.01) | -- | -.62 (<.001) | -- | -- | -- |
|  |  | Laboratory test or diagnostic procedure | 708 | 19.25 | .29 | -- | -- | -- | -- | -- | -- | -- | -7.08 (.04) | -- | -4.84 (<.001) | -- | -.58 (<.001) | -- | -- | -- |
|  |  | New condition or symptom | 741 | 2.35 | .30 | -- | -- | -- | -- | -- | -- | 3.16 (.03) | -- | -- | -4.18 (<.01) | -- | -.61 (<.001) | -- | -- | -- |
|  |  | Preventive care or physical exam | 678 | 18.77 | .30 | -- | -- | -- | -- | -- | -- | 3.43 (.03) | -- | -- | -4.22 (<.01) | -- | -.61 (<.001) | -- | -- | -- |
|  |  | Reschedule | 874 | 23.24 | .29 | -- | -- | -- | -- | -- | -- | 4.55 (<.01) | -- | -- | -4.23 (<.01) | -- | -.62 (<.001) | -.16 (<.01) | -- | -- |
| Social communication | Grouped | -- | 760 | 2.00 | .29 | -- | -- | -- | -- | -- | -- | 4.00 (<.01) | -- | -- | -4.56 (<.001) | -- | -.57 (<.001) | -- | -- | -- |
|  | Appreciation and praise | -- | 666 | 19.24 | .31 | 4.64 (.02) | -- | -- | -- | -- | -- | 3.21 (.04) | -- | -- | -4.51 (<.01) | -- | -.60 (<.001) | -- | -- | -- |
|  | Complaints | -- | 682 | 18.66 | .30 | -4.03 (.04) | -- | -- | -- | -- | -- | 3.34 (.03) | -- | -- | -4.47 (<.01) | -- | -.60 (<.001) | -- | -- | -- |
|  | Life issues | -- | 704 | 19.45 | .30 | -- | -- | -- | -- | -- | -- | 3.90 (.01) | -- | -- | -4.89 (<.001) | -- | -.58 (<.001) | -- | -- | -- |
| Action responses | Grouped | -- | 1091 | 27.02 | .27 | -- | -- | -- | -- | -- | -- | 3.70 (<.01) | -- | -- | -2.98 (.01) | -- | -.60 (<.001) | -.11 (.02) | -- | -- |
|  | Acknowledge | -- | 790 | 18.82 | .27 | -- | -- | -- | -- | -- | -- | 3.71 (.01) | -- | -- | -4.72 (<.001) | -- | -.59 (<.001) | -- | -- | -- |
|  | Fulfills request | -- | 1035 | 26.20 | .28 | -- | -- | -- | -- | -- | -- | 4.11 (<.01) | -- | -- | -3.20 (<.01) | -- | -.61 (<.001) | -.13 (.01) | -- | -- |
|  | Partially fulfills request | -- | 803 | 2.81 | .28 | -- | -- | -- | -- | -- | -- | 3.57 (.01) | -- | -- | -3.58 (<.01) | -- | -.58 (<.001) | -.12 (.04) | -- | -- |
| Information seeking | -- | -- | 949 | 21.90 | .26 | -- | -- | -- | -- | -- | -- | 4.86 (<.001) | -- | -- | -3.89 (<.01) | -- | -.59 (<.001) | -- | -- | -- |
| Information sharing | Grouped | -- | 1124 | 26.18 | .26 | -- | -- | -- | -- | -- | -- | 4.44 (<.001) | -- | -- | -3.16 (.01) | -- | -.60 (<.001) | -- | -- | -- |
|  | Deferred | -- | 935 | 24.04 | .28 | .91  (.03) | -- | -- | -- | -- | -- | 4.56 (<.001) | -- | -- | -3.78 (<.01) | -- | -.61 (<.001) | -.13 (.01) | -- | -- |
|  | Medical guidance | -- | 920 | 2.42 | .25 | -- | -- | -- | -- | -- | -- | 4.19 (<.01) | -- | -- | -3.25 (.01) | -- | -.57 (<.001) | -- | -- | -- |
|  | Orientation to procedures or treatments | -- | 1051 | 24.74 | .26 | -- | -- | -- | -- | -- | -- | 4.10 (<.01) | -- | -- | -3.74 (<.01) | -- | -.60 (<.001) | -- | -- | -- |
| Social communication | Encouragement | -- | 656 | 18.13 | .30 | -- | -- | -- | -- | -- | -- | 3.62 (.02) | -- | -- | -4.27 (<.01) | -- | -.60 (<.001) | -- | -- | -- |
| Non-response | -- | -- | 1031 | 27.88 | .29 | -- | -- | -- | -- | -- | -- | 2.81 (.02) | -- | -- | -3.50 (<.01) | -- | -.60 (<.001) | -- | -- | -- |

Notes: DBP=Diastolic blood pressure; HTN=Hypertension; OVs=Outpatient visits; SBP=Systolic blood pressure Includes patients sent or received at least one message coded with the selected taxon plus all patients who did not initiate a thread in 2017. Each row represents a linear regression model where the dependent variable is the change in SBP percentage between 2016 and 2018. The independent variable is the taxon prevalence and the covariates are the patient characteristics. -- Not significant at *P*<.05. *Non-response is not a taxon; rather, it is identified as any message thread that includes no clinic staff secure message response.

Table 3-5. Linear Regression Results Estimating Associations Between Taxa and Diastolic Blood Pressure Changes, Among the Secure Messaging Population

| **Level 1 Taxon** | **Level 2 Taxon** | **Level 3 Taxon** | **F-value** (all significant at *P*<.001) | **R-square** | **Taxon b weight (*P*-value)** | **b weight (*P*-value)** | | | | | | | | | | | | | | |
| --- | --- | --- | --- | --- | --- | --- | --- | --- | --- | --- | --- | --- | --- | --- | --- | --- | --- | --- | --- | --- |
|  |  |  |  |  |  | **Age (years)** | **Average distance to clinic** | **Male vs female** | **Other payer vs Private payer** | **Public payer vs Private payer** | **Uninsured vs Private payer** | **Black vs white** | **Other race vs white** | **Rural vs urban home location** | **Hypertension only vs Diabetes & hypertension** | **Baseline DBP** | **Baseline SBP** | **Number of outpatient visits** | **Number of inpatient visits** | **Number of threads** |
| Information seeking | Grouped | -- | 19.73 | .35 | -- | -.11 (<.01) | -- | -- | -- | -- | -- | -- | -- | -- | -- | -.69 (<.001) | -- | -.09 (<.01) | -- | -- |
|  | Logistics | -- | 19.78 | .35 | -- | -.11 (<.01) | -- | -- | -- | -- | -- | -- | -- | -- | -- | -.69 (<.001) | -- | -.09 (<.01) | -- | -- |
|  | Medical guidance | -- | 19.76 | .35 | -- | -.11 (<.01) | -- | -- | -- | -- | -- | -- | -- | -- | -- | -.69 (<.001) | -- | -.09 (<.01) | -- | -- |
| Information sharing | Grouped |  | 19.74 | .35 | -- | -.11 (<.01) | -- | -- | -- | -- | -- | -- | -- | -- | -- | -.69 (<.001) | -- | -.09 (<.01) | -- | -- |
|  | Clinical update | -- | 19.73 | .35 | -- | -.11 (<.01) | -- | -- | -- | -- | -- | -- | -- | -- | -- | -.69 (<.001) | -- | -.09 (<.01) | -- | -- |
|  | Response to clinician’s message | -- | 19.74 | .35 | -- | -.11 (<.01) | -- | -- | -- | -- | -- | -- | -- | -- | -- | -.69 (<.001) | -- | -.09 (<.01) | -- | -- |
|  | Self-reporting | -- | 19.81 | .35 | -- | -.11 (<.01) | -- | -- | -- | -- | -- | -- | -- | -- | -- | -.70 (<.001) | -- | -.09 (<.01) | -- | -- |
| Task-oriented | Prescription refills and renewals requests | -- | 19.94 | .35 | -- | -.10 (<.01) | -- | -- | -- | -- | -- | -- | -- | -- | -- | -.69 (<.001) | -- | -.09 (<.01) | -- | -- |
|  | New or change prescription request | -- | 19.81 | .35 | -- | -.11 (<.01) | -- | -- | -- | -- | -- | -- | -- | -- | -- | -.70 (<.001) | -- | -.09 (<.01) | -- | -- |
|  | Other administrative | -- | 2.03 | .35 | -- | -.10 (<.01) | -- | -- | -- | -- | -- | -- | -- | -- | -- | -.69 (<.001) | -- | -.09 (<.01) | -- | -- |
|  | Referral requests | -- | 19.88 | .35 | -- | -.11 (<.01) | -- | -- | -- | -- | -- | -- | -- | -- | -- | -.70 (<.001) | -- | -.09 (<.01) | -- | -- |
|  | Scheduling request | Cancellation | 19.96 | .35 | -- | -.11 (<.01) | -- | -- | -- | -- | -- | -- | -- | -- | -- | -.70 (<.001) | -- | -.09 (<.01) | -- | -- |
|  |  | Follow-up | 19.81 | .35 | -- | -.11\| (<.01) | -- | -- | -- | -- | -- | -- | -- | -- | -- | -.69 (<.001) | -- | -.09 (<.01) | -- | -- |
|  |  | Laboratory test or diagnostic procedure | 19.85 | .35 | -- | -.11 (<.01) | -- | -- | -- | -- | -- | -- | -- | -- | -- | -.69 (<.001) | -- | -.09 (<.01) | -- | -- |
|  |  | New condition or symptom | 2.13 | .35 | .68 (.04) | -.10 (<.01) | -- | -- | -- | -- | -- | -- | -- | -- | -- | -.69 (<.001) | -- | -.09 (.01) | -- | -- |
|  |  | Preventive care or physical exam | 19.73 | .35 | -- | -.11 (<.01) | -- | -- | -- | -- | -- | -- | -- | -- | -- | -.69 (<.001) | -- | -.09 (<.01) | -- | -- |
|  |  | Reschedule | 2.34 | .36 | .44 (.01) | -.10 (<.01) | -- | -- | -- | -- | -- | -- | -- | -- | -- | -.69 (<.001) | -- | -.09 (<.01) | -- | -- |
| Social communication | Grouped | -- | 19.73 | .35 | -- | -.11 (<.01) | -- | -- | -- | -- | -- | -- | -- | -- | -- | -.69 (<.001) | -- | -.09 (<.01) | -- | -- |
|  | Appreciation and praise | -- | 2.34 | .36 | 3.21 (.01) | -.11 (<.01) | -- | -- | -- | -- | -- | -- | -- | -- | -- | -.69 (<.001) | -- | -.09 (<.01) | -- | -- |
|  | Complaints | -- | 19.80 | .35 | -- | -.11 (<.01) | -- | -- | -- | -- | -- | -- | -- | -- | -- | -.69 (<.001) | -- | -.09 (<.01) | -- | -- |
|  | Life issues | -- | 19.88 | .35 | -- | -.11 (<.01) | -- | -- | -- | -- | -- | -- | -- | -- | -- | -.69 (<.001) | -- | -.09 (<.01) | -- | -- |
| Action responses | Grouped |  | 2.16 | .35 | -.30 (.03) | -.12 (<.01) | -- | -- | -- | -- | -- | -- | -- | -- | -- | -.71 (<.001) | -- | -.11 (<.01) | -- | -- |
|  | Acknowledge | -- | 19.93 | .35 | -- | -.11 (<.01) | -- | -- | -- | -- | -- | -- | -- | -- | -- | -.69 (<.001) | -- | -.10 (<.01) | -- | -- |
|  | Fulfills request | -- | 19.85 | .35 | -- | -.11 (<.01) | -- | -- | -- | -- | -- | -- | -- | -- | -- | -.70 (<.001) | -- | -.10 (<.01) | -- | -- |
|  | Partially fulfills request | -- | 19.88 | .35 | -- | -.11 (<.01) | -- | -- | -- | -- | -- | -- | -- | -- | -- | -.70 (<.001) | -- | -.10 (<.01) | -- | -- |
| Information seeking | -- | -- | 19.92 | .35 | -- | -.11 (<.01) | -- | -- | -- | -- | -- | -- | -- | -- | -- | -.69 (<.001) | -- | -.10 (<.01) | -- | -- |
| Information sharing | Grouped | -- | 2.02 | .35 | -- | -.12 (<.01) | -- | -- | -- | -- | -- | -- | -- | -- | -- | -.70 (<.001) | -- | -.10 (<.01) | -- | -- |
|  | Deferred | -- | 2.09 | .35 | -- | -.11 (<.01) | -- | -- | -- | -- | -- | -- | -- | -- | -- | -.69 (<.001) | -- | -.10 (<.01) | -- | -- |
|  | Medical guidance | -- | 19.75 | .35 | -- | -.11 (<.01) | -- | -- | -- | -- | -- | -- | -- | -- | -- | -.69 (<.001) | -- | -.09 (<.01) | -- | -- |
|  | Orientation to procedures or treatments | -- | 2.33 | .36 | .47 (.01) | -.11 (<.01) | -- | -- | -- | -- | -- | -- | -- | -- | -- | -.70 (<.001) | -- | -.10 (<.01) | -- | -- |
| Social communication | Encouragement | -- | 19.87 | .35 | -- | -.11 (<.01) | -- | -- | -- | -- | -- | -- | -- | -- | -- | -.69 (<.001) | -- | -.09 (<.01) | -- | -- |
| Non-response | -- | -- | 2.21 | .35 | -.30 (.02) | -.10 (<.01) | -- | -- | -- | -- | -- | -- | -- | -- | -- | -.69 (<.001) | -- | -.09 (.01) | -- | -- |

Notes: N=607. DBP=Diastolic blood pressure; SBP=Systolic blood pressure. Includes only patients who initiated at least one secure message thread in 2017. Each row represents a linear regression model where the dependent variable is the change in DBP percentage between 2016 and 2018. The independent variable is the taxon prevalence and the covariates are the patient characteristics. -- Not significant at *P*<.05. *Non-response is not a taxon; rather, it is identified as any message thread that includes no clinic staff secure message response.

Table 3-6. Linear Regression Results Estimating Associations Between Taxa and Diastolic Blood Pressure Changes, Among the Full Population

| **Level 1 Taxon** | **Level 2 Taxon** | **Level 3 Taxon** | **N** | **F-value** (all significant at *P*<.001) | **R-square** | **Taxon b weight (*P*-value)** | **b weight (*P*-value)** | | | | | | | | | | | | | |
| --- | --- | --- | --- | --- | --- | --- | --- | --- | --- | --- | --- | --- | --- | --- | --- | --- | --- | --- | --- | --- |
|  |  |  |  |  |  |  | **Age (years)** | **Male vs female** | **Other payer vs Private payer** | **Public payer vs Private payer** | **Uninsured vs Private payer** | **Black vs white** | **Other race vs white** | **Rural vs urban home location** | **HTN only vs Both** | **Baseline DBP** | **Baseline SBP** | **Number of outpatient visits** | **Number of inpatient visits** | **Number of threads** |
| Information seeking | Grouped | -- | 1022 | 36.94 | .36 | -- | -.14 (<.001) | -- | -- | -- | -- | 1.89 (<.01) | -- | -- | 1.33 (.03) | -.64 (<.001) | -- | -.06 (.03) | -- | -- |
|  | Logistics | -- | 885 | 33.35 | .37 | -- | -.15 (<.001) | -- | -- | -- | -- | 2.26 (<.001) | -- | -- | -- | -.64 (<.001) | -- | -.06 (.02) | -- | -- |
|  | Medical guidance | -- | 965 | 34.66 | .35 | -- | -.14 (<.001) | -- | -- | -- | -- | 1.98 (<.01) | -- | -- | 1.57 (.01) | -.64 (<.001) | -- | -.06 (.02) | -- | -- |
| Information sharing | Grouped | -- | 1034 | 37.28 | .35 | -- | -.12 (<.001) | -- | -- | -- | -- | 2.38 (<.001) | -- | -- | 1.37 (.02) | -.65 (<.001) | -- | -.05 (.03) | -- | -- |
|  | Clinical update | -- | 909 | 34.34 | .37 | -- | -.12 (<.001) | -- | -- | -1.76 (.04) | -- | 2.07 (<.01) | -- | -- | 1.70 (<.01) | -.65 (<.001) | -- | -- | -- | -- |
|  | Response to clinician’s message | -- | 937 | 34.53 | .36 | -- | -.13 (<.001) | -- | -- | -- | -- | 2.45 (<.001) | -- | -- | -- | -.65 (<.001) | -- | -.07 (<.01) | -- | -- |
|  | Self-reporting | -- | 684 | 26.51 | .37 | -- | -.15 (<.001) | -- | -- | -- | -- | 2.92 (<.001) | -- | -- | 1.70 (.02) | -.65 (<.001) | -- | -- | -- | -- |
| Task-oriented | Prescription refills and renewals requests | -- | 925 | 33.53 | .36 | -- | -.13 (<.001) | -- | -- | -1.73 (.04) | -- | 2.21 (<.001) | -- | -- | -- | -.63 (<.001) | -- | -- | -- | -- |
|  | New or change prescription request | -- | 784 | 27.94 | .35 | -- | -.13 (<.001) | -- | -2.34 (.02) | -2.62 (<.01) | -- | 2.46 (<.01) | -- | -- | 1.45 (.04) | -.62 (<.001) | -- | -- | -- | -- |
|  | Other administrative | -- | 806 | 33.01 | .39 | -- | -.16 (<.001) | -- | -2.37 (.02) | -2.14 (.02) | -- | 1.98 (<.01) | -- | -- | -- | -.64 (<.001) | -- | -- | -- | -- |
|  | Referral requests | -- | 692 | 27.04 | .38 | -- | -.13 (<.001) | -- | -2.32 (.03) | -2.41 (.02) | -- | 2.53 (<.01) | -- | -- | -- | -.63 (<.001) | -- | -- | -- | -- |
|  | Scheduling request | Cancellation | 757 | 28.87 | .37 | -- | -.14 (<.001) | -- | -2.19 (.03) | -- | -- | 2.75 (<.001) | -- | -- | -- | -.64 (<.001) | -- | -.07 (.03) | -- | -- |
|  |  | Follow-up | 764 | 28.22 | .36 | -- | -.13 (<.001) | -- | -- | -- | -- | 2.43 (<.001) | -- | -- | -- | -.62 (<.001) | -- | -.06 (.02) | -- | -- |
|  |  | Laboratory test or diagnostic procedure | 708 | 28.21 | .38 | -- | -.16 (<.001) | -- | -2.47 (.02) | -- | -- | 2.38 (<.01) | -- | -- | -- | -.63 (<.001) | -- | -- | -- | -- |
|  |  | New condition or symptom | 741 | 28.94 | .37 | -- | -.14 (<.001) | -- | -- | -- | -- | 2.35 (<.01) | -- | -- | -- | -.62 (<.001) | -- | -- | -- | -- |
|  |  | Preventive care or physical exam | 678 | 26.42 | .37 | -- | -.15 (<.001) | -- | -2.47 (.02) | -2.11 (.04) | -- | 2.49 (<.001) | -- | -- | 1.48 (.04) | -.61 (<.001) | -- | -.07 (.02) | -- | -- |
|  |  | Reschedule | 874 | 33.05 | .37 | .33  (.04) | -.14 (<.001) | -- | -- | -- | -- | 2.91 (<.001) | -- | -- | -- | -.65 (<.001) | -- | -.07 (.01) | -- | -- |
| Social communication | Grouped | -- | 760 | 31.08 | .39 | -- | -.15 (<.001) | -- | -2.06 (.04) | -- | -- | 2.52 (<.001) | -- | -- | -- | -.63 (<.001) | -- | -- | -- | -- |
|  | Appreciation and praise | -- | 666 | 27.86 | .39 | 2.60 (.01) | -.16 (<.001) | -- | -2.63 (.02) | -- | -- | 2.40 (<.01) | -- | -- | -- | -.63 (<.001) | -- | -- | -- | -- |
|  | Complaints | -- | 682 | 26.66 | .38 | -- | -.16 (<.001) | -- | -2.71 (.01) | -- | -- | 2.54 (<.01) | -- | -- | -- | -.61 (<.001) | -- | -- | -- | -- |
|  | Life issues | -- | 704 | 29.59 | .39 | -- | -.16 (<.001) | -- | -- | -- | -- | 2.57 (<.001) | -- | -- | -- | -.63 (<.001) | -- | -- | -- | -- |
| Action responses | Grouped | -- | 1091 | 38.97 | .35 | -- | -.13 (<.001) | -- | -- | -1.72 (.03) | -- | 2.09 (<.001) | -- | -- | 1.16  (.04) | -.64 (<.001) | -- | -.07 (.01) | -- | -- |
|  | Acknowledge | -- | 790 | 3.02 | .37 | -- | -.15 (<.001) | -- | -2.24 (.02) | -- | -- | 2.45 (<.001) | -- | -- | 1.54 (.02) | -.64 (<.001) | -- | -- | -- | -- |
|  | Fulfills request | -- | 1035 | 36.83 | .35 | -- | -.13 (<.001) | -- | -- | -1.72 (.03) | -- | 2.40 (<.001) | -- | -- | 1.19 (.04) | -.63 (<.001) | -- | -.08 (<.01) | -- | -- |
|  | Partially fulfills request | -- | 803 | 3.13 | .36 | -- | -.13 (<.001) | -- | -- | -- | -- | 2.21 (<.01) | -- | -- | -- | -.64 (<.001) | -- | -.07 (.01) | -- | -- |
| Information seeking | -- | -- | 949 | 34.18 | .35 | -- | -.13 (<.001) | -- | -- | -- | -- | 2.61 (<.01) | -- | -- | -- | -.64 (<.001) | -- | -.07 (.01) | -- | -- |
| Information sharing | Grouped | -- | 1124 | 38.92 | .35 | -- | -.12 (<.001) | -- | -- | -- | -- | 2.51 (<.001) | -- | -- | 1.39 (.02) | -.65 (<.001) | -- | -.06 (.01) | -- | -- |
|  | Deferred | -- | 935 | 34.76 | .36 | -- | -.13 (<.001) | -- | -- | -- | -- | 2.62 (<.001) | -- | -- | 1.48 (.02) | -.64 (<.001) | -- | -.08 (<.01) | -- | -- |
|  | Medical guidance | -- | 920 | 33.37 | .36 | -- | -.14 (<.001) | -- | -- | -- | -- | 2.18 (<.01) | -- | -- | 1.54 (.02) | -.65 (<.001) | -- | -- | -- | -- |
|  | Orientation to procedures or treatments | -- | 1051 | 38.27 | .36 | -- | -.13 (<.001) | -- | -- | -- | -- | 2.44 (<.001) | -- | -- | -- | -.66 (<.001) | -- | -.06 (.02) | -- | -- |
| Social communication | Encouragement | -- | 656 | 25.88 | .38 | -- | -.16 (<.001) | -- | -2.64 (.02) | -- | -- | 2.66 (<.001) | -- | -- | -- | -.63 (<.001) | -- | -- | -- | -- |
| Non-response* | -- | -- | 1031 | 37.02 | .35 | -.23 (.04) | -.13 (<.001) | -- | -- | -- | -- | 1.70 (<.01) | -- | -- | -- | -.63 <.001 | -- | -.07 (.01) | -- | -- |

Notes: DBP=Diastolic blood pressure; HTN=Hypertension; SBP=Systolic blood pressure. Includes patients sent or received at least one message coded with the selected taxon plus all patients who did not initiate a thread in 2017. Each row represents a linear regression model where the dependent variable is the change in DBP percentage between 2016 and 2018. The independent variable is the taxon prevalence and the covariates are the patient characteristics. -- Not significant at *P*<.05. *Non-response is not a taxon; rather, it is identified as any message thread that includes no clinic staff secure message response.
